# CDC-42 Orients Cell Migration during Epithelial Intercalation in the *Caenorhabditis elegans* Epidermis

**DOI:** 10.1371/journal.pgen.1006415

**Published:** 2016-11-18

**Authors:** Elise Walck-Shannon, Bethany Lucas, Ian Chin-Sang, David Reiner, Kraig Kumfer, Hunter Cochran, William Bothfeld, Jeff Hardin

**Affiliations:** 1 Department of Zoology, University of Wisconsin-Madison, Madison, Wisconsin, United States of America; 2 Program in Genetics, University of Wisconsin-Madison, Madison, Wisconsin, United States of America; 3 Department of Biology, Queen's University, Kingston, Ontario, Canada; 4 Center for Translational Cancer Research, Institute of Biosciences and Technology and Department of Medical Physiology, Texas A&M Health Science Center, Houston, Texas, United States of America; 5 Department of Medicine, University of Wisconsin-Madison, Madison, Wisconsin, United States of America; University of Kansas, UNITED STATES

## Abstract

Cell intercalation is a highly directed cell rearrangement that is essential for animal morphogenesis. As such, intercalation requires orchestration of cell polarity across the plane of the tissue. CDC-42 is a Rho family GTPase with key functions in cell polarity, yet its role during epithelial intercalation has not been established because its roles early in embryogenesis have historically made it difficult to study. To circumvent these early requirements, in this paper we use tissue-specific and conditional loss-of-function approaches to identify a role for CDC-42 during intercalation of the *Caenorhabditis elegans* dorsal embryonic epidermis. CDC-42 activity is enriched in the medial tips of intercalating cells, which extend as cells migrate past one another. Moreover, CDC-42 is involved in both the efficient formation and orientation of cell tips during cell rearrangement. Using conditional loss-of-function we also show that the PAR complex functions in tip formation and orientation. Additionally, we find that the sole *C*. *elegans* Eph receptor, VAB-1, functions during this process in an Ephrin-independent manner. Using epistasis analysis, we find that *vab-1* lies in the same genetic pathway as *cdc-42* and is responsible for polarizing CDC-42 activity to the medial tip. Together, these data establish a previously uncharacterized role for polarized CDC-42, in conjunction with PAR-6, PAR-3 and an Eph receptor, during epithelial intercalation.

## Author Summary

As embryos develop, tissues must change shape to establish an animal’s form. One key form-shaping movement, cell intercalation, often occurs when a tissue elongates in a preferred direction. How cells in epithelial sheets can intercalate while maintaining tissue integrity is not well understood. Here we use the dorsal epidermis in embryos of the nematode worm, *C*. *elegans*, to study cell intercalation. As cells begin to intercalate, they form highly polarized tips that lead their migration. While some mechanisms that polarize intercalating cells have been established in other systems, our work identifies a new role for CDC-42—a highly conserved, highly regulated protein that controls the actin cytoskeleton. We previously established that a related protein, Rac, is involved in tip extension during dorsal intercalation. CDC-42 also contributes to this process in addition to helping orient the extending tip. CDC-42 appears to work in conjunction with two other known cell polarity proteins, PAR-3 and PAR-6, and the cell surface receptor, VAB-1. Our work identifies a novel pathway involving proteins conserved from worms to humans that regulates a ubiquitous process during animal development.

## Introduction

Understanding how the cellular behaviors that underlie embryonic morphogenesis are regulated is a longstanding and fundamental goal of developmental biology. One such cell behavior that can reshape tissues during embryogenesis is mediolateral intercalation, whereby cells within the same plane interdigitate along a preferred axis (reviewed in [1]). Mediolateral intercalation is a widespread process in animal development; it occurs in tissues derived from all three germ layers during gastrulation and organogenesis. There are multiple mechanisms by which cells can intercalate, including highly directed and coordinated protrusive activity and highly polarized apical junction rearrangement (reviewed in [1]). These mechanisms are unified by a fundamental theme: movement must occur in a highly polarized fashion.

One well studied regulator of cell polarity is the Rho family GTPase Cdc42 (reviewed in [2]). As a GTPase, Cdc42 cycles between active, GTP-bound and inactive, GDP-bound states. Cdc42-GTP has multiple molecular functions that allow it to influence cell polarity. Active Cdc42 can modify the actin cytoskeleton to produce long, thin filopodia [3], a process mediated through its interaction with the actin nucleation promoting factor **W**iskott-**A**lrich **s**yndrome **p**rotein (WASP) [4]. Furthermore, through interactions with the **par**titioning defective (PAR) complex [5–7], Cdc42 can regulate the microtubule cytoskeleton to reorient centrosomes in order to polarize cell division and regulate apical junctions in epithelia [8, 9]. Given these diverse and conserved molecular processes, it is reasonable to think that Cdc42 has roles in polarizing epithelia during intercalation.

While Cdc42 has documented roles in other intercalation events, it has not yet been implicated during embryonic epithelial morphogenesis. For example, Cdc42 helps orient intercalation in frog mesenchymal cells during convergent extension through the planar cell polarity pathway [10], in the ascidian notochord during convergent extension as dorsoventral polarity is established [11], in the fly mesoderm during gastrulation through fibroblast growth factor signaling [12], and during transendothelial migration as cancer cells metastasize [13]. While Cdc42 has a documented role in orienting cell divisions in an intercalating epithelium, the frog neuroepithelium [14], no role has been documented for Cdc42 in the rearrangement of post-mitotic intercalating cells.

To study Cdc42 in an intercalating epithelium, we employed the *Caenorhabditis elegans* dorsal epidermis. During dorsal intercalation, two rows of ten epidermal cells interdigitate into a single row of twenty [15]. Dorsal intercalation is accompanied by the appearance of highly protrusive medial tips; in contrast, lateral (i.e., rear) cell borders are protrusively inactive [16]. Previous work made CDC-42 a good candidate for regulating polarity during dorsal intercalation. CDC-42 has roles in collective cell migration of ventral epidermal cells in the *C*. *elegans* embryo [17], suggesting that it is active in the epidermis. Moreover, we recently discovered that *wsp-1*/WASP, a key Cdc42 downstream effector, and *wve-1*/WAVE function redundantly to promote medial protrusive activity during dorsal intercalation [16].

In this study, we use a combination of two genetic approaches—epidermal-specific dominant-negative mutant CDC-42 and late loss of CDC-42 function—along with *in vivo* CDC-42 biosensor data to establish a role for CDC-42 in forming and fine-tuning the orientation of the medial tips of dorsal epidermal cells as they extend during intercalation. Additionally, we leverage results from large-scale genetic synthetic lethal screens [18] to implicate VAB-1/**Eph R**eceptor (EphR) as an upstream activator of CDC-42 in this context. Together, these results demonstrate the importance of CDC-42 as a key regulator of oriented cell migration and identify a genetic pathway involving highly conserved molecular components centered on CDC-42 that operates during epithelial cell intercalation.

## Results

### CDC-42 is required for the intercalation of dorsal epidermal cells

Cdc42 is a Rho family GTPase that polarizes cells during migration [19]. Previously, examination of the role of CDC-42 in cellular migrations in intact *C*. *elegans* embryos has been hampered by early requirements during anterior-posterior axis formation [6, 20]. To determine if CDC-42 function is required for the highly directed cell migrations that occur during dorsal intercalation (about 3 hours after the first division of the zygote), we sought to bypass these early requirements. To do so, we generated an epidermal-specific, inducible dominant-negative CDC-42(T17N) [21] (hereafter *cdc-42(DN)*) using the **N**onsense **M**ediated **D**ecay (NMD)-mediated system that we described previously for CED-10/Rac [16]. Briefly, transgenes expressing cDNA under control of the epidermal-specific *lin-26* promoter and an NMD-sensitive 3’UTR are generated in the temperature-sensitive *smg-1(cc546*ts*)* (NMD defective) background; at the permissive temperature of 15°C, transcripts from these transgenes are degraded by the NMD RNA surveillance system. Conversely, at the restrictive temperature of 25°C the NMD system is inactivated, transcripts from the transgenes are stabilized, and protein is produced. In addition, we utilize a second, previously established transgene-mediated system to conditionally perturb CDC-42 and PAR-3/PAR-6 function. This system relies on the degradation of functional ZF1-tagged transgenes [22–24] in mutant backgrounds, which rescue the mutant phenotype in the early embryo, thus circumventing requirements [6, 20], but lead to loss of function during gastrulation and later in embryogenesis.

**Fig 1:**
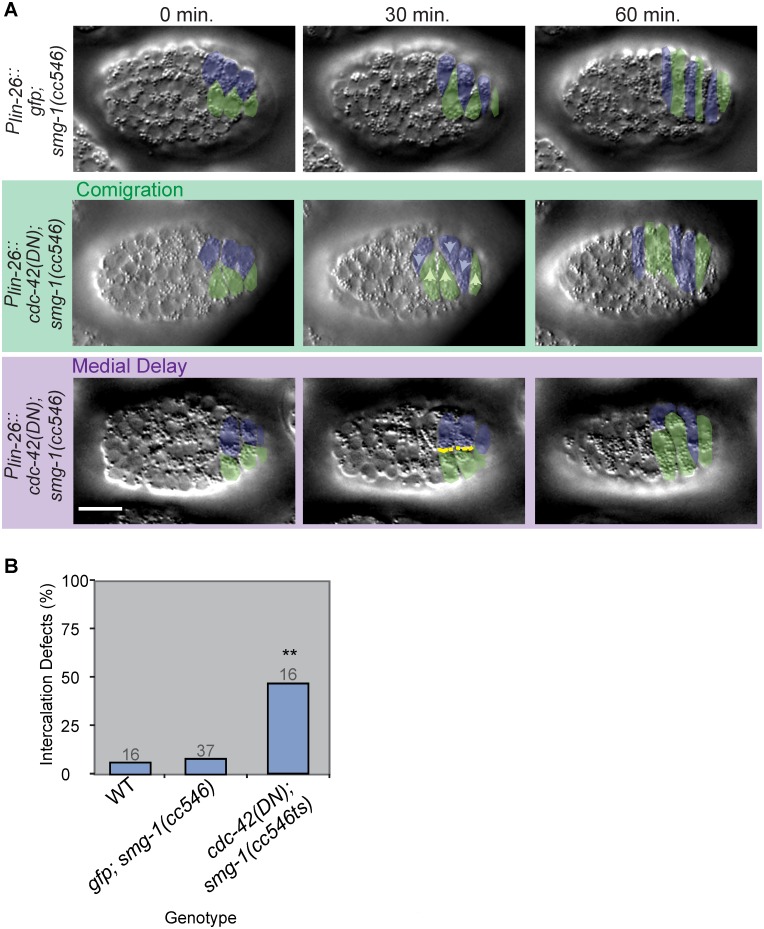
*cdc-42* loss of function phenotypes during dorsal intercalation. A) DIC micrographs of *P*_*lin-26*_::*gfp; smg-1(cc546)* (top) and two different *P*_*lin-26*_::*cdc-42(DN); smg-1(cc546)* embryos. Left dorsal cells pseudocolored green, right cells pseudocolored blue. Arrowheads indicate direction of migration; yellow dotted lines outline blunt medial edges. Dorsal intercalation time measurements in Figs 1 and 2 were performed as in [16]: 0 min. was defined as 60 min. after terminal epidermal (Cpaa.a/p) divisions. Scale bar = 10 μm. Embryos incubated at 25°C for 24 hours prior to mounting. B) Penetrance of combined ipsilateral comigration and medial delay phenotypes in wild-type (WT), *P*_*lin-26*_::*gfp; smg-1(cc546)*, and *P*_*lin-26*_::*cdc-42(DN); smg-1(cc546)* embryos. All embryos imaged at 25°C. Total defects in *cdc-42(DN*) and *ZF1*::*cdc-42* embryos (see Fig 2) were significantly different from controls (p≤0.01; **) but not each other (p = 0.749) (Fisher’s Exact Test). Gray numbers indicate number of embryos analyzed per genotype.

Upon incubation at 25°C, *cdc-42(DN)* embryos displayed two classes of intercalation defects, which were seen at significantly higher frequencies than in wild-type or in *gfp* expressing controls (p≤0.01, Fisher’s Exact Test) (Fig 1). First, adjacent dorsal cells often migrated together across the dorsal array, instead of interdigitating with contralateral neighbors (40%, n = 16), a phenotype we call “ipsilateral comigration”. This defect may be due to a mispolarization of the extending tip during intercalation. Second, medial edges of dorsal cells occasionally appeared blunt, rather than extending a pointed tip (7%, n = 16), as if opposing cells mutually block migration of its contralateral partner. We have described this phenotype previously [16]; here we refer to it as “medial delay”. These defects may result from a weak requirement for CDC-42 activity in early tip formation. Both types of defects sometimes occurred within the same pair of cells (i.e., cells tips are blunt, then migrate in the wrong direction); Fig 1B accordingly shows the pooled frequency of these two defects in *cdc-42* loss of function embryos.

**Fig 2:**
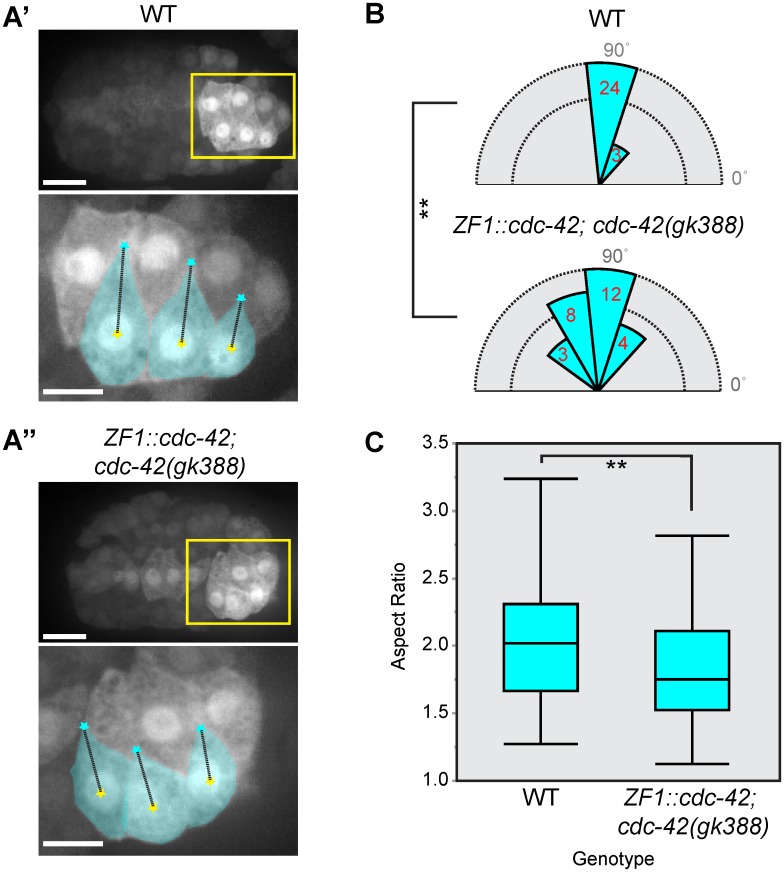
Cell morphology in *cdc-4*2 loss of function embryos. A) Confocal images of an epidermal cytoplasmic reporter (P_lbp-1_::GFP) in wild-type (WT) (A’) and *ZF1*::*cdc-42; cdc-42(gk388)* (A”) embryos, in which there is late maternal loss of CDC-42, 45 min. after terminal division. Yellow box outlines area enlarged in bottom image. Left cells pseudocolored cyan. Black dotted line connects tip (cyan star) and cell centroid (yellow star), which is quantified in B. Scale bars: top = 10 μm; bottom = 5 μm. B) Tip angle in wild-type and *ZF1*::*cdc-42; cdc-42(gk388)* cells. The distribution of tip angles is significantly less uniform in *ZF1*::*cdc-42; cdc-42(gk388)* than WT (Mardia-Watson-Wheeler, p = 0.002, **). Red text indicates number of cells. C) Box plot of aspect ratio in wild-type (n = 32) and ZF1::*cdc*-42 (n = 27) cells. Age-matched *ZF1*::*cdc-42; cdc-42(gk388)* cells were significantly less elongated than wild-type (Student’s T-test, p = 0.024, **).

To verify that these defects were due to reduction of CDC-42 function, we utilized a second approach to circumvent early requirements for CDC-42. Transgenic CDC-42 tagged with the ZF1 degron is stable during initial zygotic divisions but sensitive to degradation by the E3 ubiquitin ligase ZIF-1 during gastrulation in somatic cells, leading to late maternal loss of function [22, 23]. The ZF1::CDC-42-expressing transgene can be combined with the putative null allele, *cdc-42(gk388)* [25, 26] to additionally mediate strong zygotic loss of CDC-42 function. The resulting *ZF1*::*cdc-42; cdc-42(gk388)* embryos (hereafter *ZF1*::*cdc-*42) [23, 27] circumvent early requirements for CDC-42 while depleting CDC-42 in the later embryo. Both ipsilateral comigration and medial delay occurred in *ZF1*::*cdc-42* embryos (20% and 24%, respectively, n = 35; Fig 2), and the penetrance of these defects was not significantly different from *cdc-42(DN)* embryos (p = 0.749, Fisher’s Exact Test). Together, these data suggest that CDC-42 has roles in both tip extension and orientation during dorsal intercalation in *C*. *elegans*.

### CDC-42 is required for medial tip orientation and extension in intercalating cells

**Fig 3:**
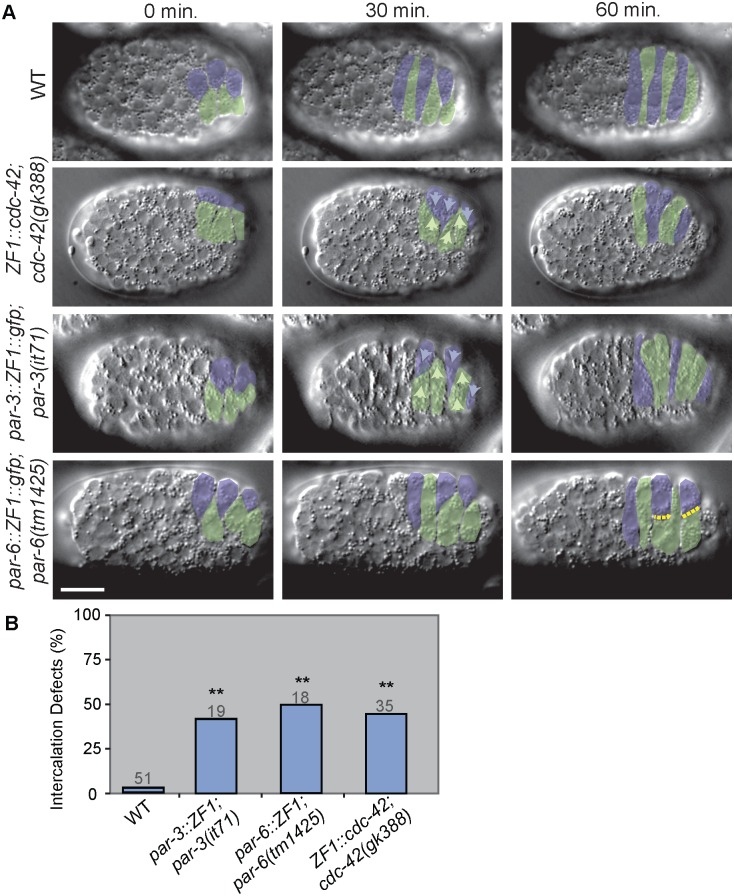
PAR complex loss of function phenotypes during dorsal intercalation. A) DIC images of wild-type (WT) and representative *par-6*::*ZF1; par-6(tm1425)*, *par-3*::*ZF1; par-3(it71)*, and *ZF1*::*cdc-42; cdc-42(gk388)* embryos. Left dorsal cells pseudocolored green, right cells pseudocolored blue. 0 min. was defined as 60 min. after terminal epidermal (Cpaa.a/p) divisions. Light arrows indicate direction of migration, while yellow dotted lines outline blunt medial edges. Scale bar = 10 μm. Embryos maintained at 20°C. B) Distribution of intercalation phenotypes in WT, *ZF1*::*cdc-42; cdc-42(gk388)*, *par-3*::*ZF1; par-3(it71)*, and *par-6*::*ZF1; par-6(tm1425)*, embryos. Total defects in *par-3*::*ZF1; par-3(it71)*, *par-6*::*ZF1; par-6(tm1425)*, and *ZF1*::*cdc-42; cdc-42(gk388)* embryos were significantly different from wild-type controls (p<0.01, **) but not each other (p≥0.7) (Fisher’s Exact Test). Gray numbers indicate number of embryos analyzed per genotype.

We next sought to determine whether atypical cell morphology preceded the cell migration defects visible by DIC microscopy in cells with compromised CDC-42. To do so, we analyzed wild-type and *ZF1*::*cdc-42* cells expressing an epidermal cytoplasmic reporter (*P*_*lbp-1*_::*gfp*) [28] at a time prior to migration but when cells normally begin to take on a mediolaterally polarized morphology (45 min. after the terminal division; for comparison, the “0 min.” time point on all DIC images is 60 min. after the terminal epidermal divisions). Our analysis uncovered two abnormalities. First, we found that the angular orientation of medial tips was significantly less uniform in *ZF1*::*cdc-42* than in age-matched wild-type controls (Mardia-Watson-Wheeler, p = 0.002; Fig 3A and 3B). In *ZF1*::*cdc-42* cells, the tip was often oriented more anteriorly or posteriorly than in wild-type, suggesting that CDC-42 is involved in fine-tuning medial tip orientation. This initial misorientation also provides a potential explanation for ipsilateral comigration: if sufficiently misoriented, once cell migration ensues, then comigration could occur.

In addition to medial tip orientation defects, our analysis revealed a second abnormality. The length/width ratio (aspect ratio) of intercalating cells was significantly lower in *ZF1*::*cdc-42* than in age-matched wild-type controls (Student’s T-test, p = 0.02; Fig 3C). *ZF1*::*cdc-42* cells were often less elongated, suggesting that CDC-42 is required for cells to properly extend their medial tips during dorsal intercalation. Such extension defects may, in severe cases, result in the medial delay phenotype observed by DIC microscopy.

Since we had shown previously that CED-10/Rac is important for tip extension during dorsal intercalation [16], we next examined whether CED-10 and CDC-42 act together in this process. When we crossed the *ced-10(n3246)* missense mutation into the *P*_*lin-26*_::*cdc-42(DN)*::*smgS; smg-1(cc546*ts*)* background, the resulting heat shocked embryos had fully penetrant intercalation defects (S1 Fig). The penetrance of total defects was significantly higher (Student’s T-test, p<0.01) than similarly heat-shocked *smg-1(cc546*ts*)*, *ced-10(n3246); smg-1(cc546*ts*)* or *P*_*lin-26*_::*cdc-42(DN)*::*smgS; smg-1(cc546*ts*)* controls. Significantly, *P*_*lin-26*_::*cdc-42(DN)*::*smgS; ced-10(n3246); smg-1(cc546*ts*)* loss-of-function embryos had a significantly greater penetrance of medial delay than controls (ANOVA, p<0.02), while the penetrance of ipsilateral comigration remained the same, suggesting that CED-10/Rac and CDC-42 normally cooperate specifically to extend medial tips. Taken together, these data support a minor role for CDC-42 in tip extension during dorsal intercalation.

### *par-6* and *par-3* are also required for dorsal intercalation

Cdc42 forms a complex with Par6 and Par3 to influence cell polarity [7], so we next compared the phenotypes of *cdc-42* and *par* loss of function during dorsal intercalation. We again utilized existing ZF1 transgenes to abrogate *par-3* and *par-6* function [8, 9, 24]. Late maternal loss of function in *par-3*::*ZF1*::*gfp; par-3(it71)* embryos led to defects closely resembling those in *ZF1*::*cdc-42* and *cdc-42(DN)* embryos, including ipsilateral comigration and medial delay (37% and 5%, respectively, n = 19) (Fig 3). Late maternal *par-6* loss of function—through *par-6*::*ZF1*::*gfp* combined with zygotic loss of function, using the strong loss of function *par-6(tm1425)* allele—resulted in similar phenotypes (33% ipsilateral comigration and 17% medial delay, n = 18) (Fig 3). Together, these results suggest that CDC-42 and the PAR complex both act to orient and extend medial tips during dorsal intercalation.

### CDC-42 activity is enriched at the medial tip

**Fig 4:**
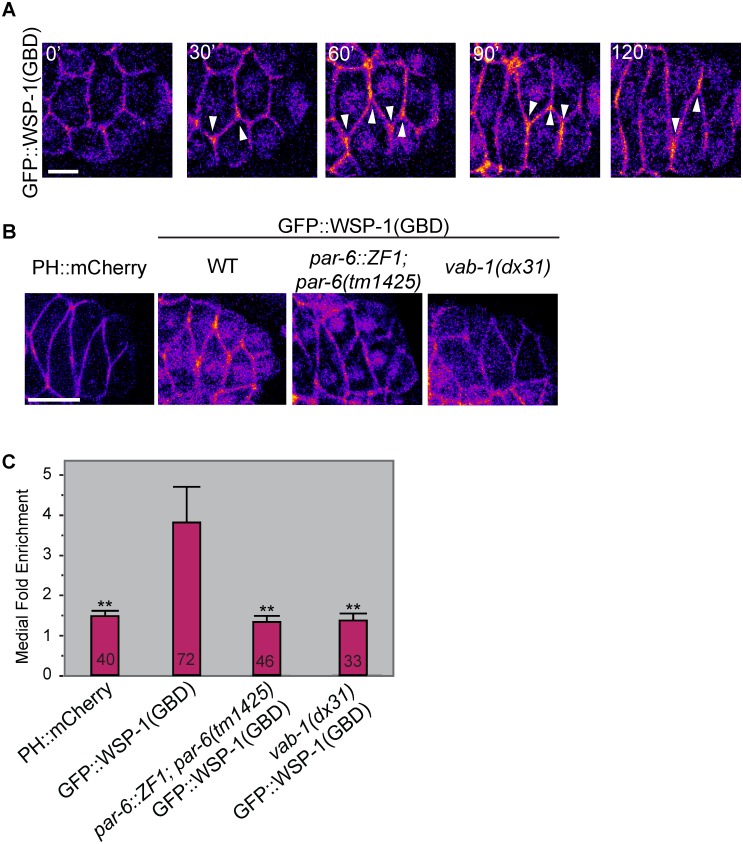
Localization of a CDC-42 biosensor (GFP::WSP-1(**G**-protein **b**inding **d**omain[GBD])) during dorsal intercalation A) in wild-type embryos. Micrographs pseudocolored according to fluorescence intensity using the “Fire” lookup table in ImageJ. As the medial edge becomes pointed, it accumulates active CDC-42 (white arrows), which is maintained during intercalation. Scale bar = 5 μm. B) Comparison of PH::mCherry in wild-type embryos and CDC-42 biosensor localization in *par-6*::*ZF1*, *vab-1*, and otherwise wild-type embryos during dorsal intercalation as in A). Scale bar = 10 μm. C) Quantification of the medial versus lateral enrichment of active CDC-42 in various genotypes. Medial enrichment was significantly greater for GFP::WSP-1(GBD) than PH::mCherry controls (Student’s T-Test, p = 0.01; **). Medial enrichment of GFP::WSP-1(GBD) was significantly decreased in both the *par-6*::*ZF1; par-6(tm1425)* and *vab-1(dx31)* backgrounds (Student’s T-Test, p≤0.01; **). Numbers indicate number of cells analyzed per genotype.

If CDC-42 is involved in medial tip formation, it might be expected that its activity would be enriched at sites of tip formation. As a Rho family GTPase, CDC-42 cycles between active (GTP-bound) and inactive (GDP-bound) states. We determined the localization of active CDC-42 using a validated CDC-42 biosensor (*wsp-1(G-protein binding domain)*::*gfp* [29]), under the control of its endogenous promoter (hereafter *wsp-1(GBD)*::*gfp)*. WSP-1(GBD)::GFP localization was significantly enriched at the medial edge relative to a uniformly distributed membrane marker, PH::mCherry [30] (Student’s T-test, p = 0.03, Fig 4C). WSP-1(GBD)::GFP localization was polarized from the time of tip formation (about 30–60 min. post-terminal division) through tip extension (about 90–120 min. post-terminal division; Fig 4A). The timing of this medial enrichment of active CDC-42 coincides with the timing of tip orientation and extension, and with the defects in intercalation we observed in *cdc-42* loss of function backgrounds described above (see Fig 2).

We next addressed the functional relationship between CDC-42 and the PAR proteins during dorsal intercalation. Construction of strains for standard epistasis testing proved technically challenging due to maternal requirements for CDC-42, PAR-3, and PAR-6, and complexities in genotyping, and we were unable to recover transgenic lines following injection of DNA encoding the CDC-42 activity biosensor into *par-3*::*ZF1*::*gfp; par-3(it71)* worms. However, we were able to obtain one line of *par-6*::*ZF1*::*gfp*;*par-6(tm1425)* worms expressing *wsp-1(GBD)*::*gfp*. Based on the previous literature, there were two possible predicted outcomes from this experiment. Since PAR-6 is typically considered a downstream effector of Cdc42 [2], loss of *par-6* function might be predicted to have little effect on CDC-42 activity. However, a study in *Drosophila* neuroblasts [31] and results from the one-cell *C*. *elegans* zygote [29] (reviewed in [32]) suggested another possibility. These studies indicated positive feedback between CDC-42 and PAR-6. In this case, loss of *par-6* activity might be expected to lead to loss of CDC-42 activity at the midline due to disruption of the feedback loop. As Fig 4B and 4C indicates, *par-6*::*ZF1*::*gfp*;*par-6(tm1425)* embryos show loss of accumulation of WSP-1(GBD)::GFP at the dorsal midline, suggesting that CDC-42 and PAR-6 may engage in positive feedback to polarize intercalating cells in the dorsal epidermis.

### VAB-1/EphR is required for medial enrichment of CDC-42 activity

We next sought to identify additional components of the CDC-42 pathway that regulates dorsal intercalation. To do so, we mined the literature for genes that might be associated with activation of CDC-42. We identified one gene expressed in the dorsal epidermis during intercalation, *vab-1*/*EphR* [33], which had a genetic interaction with *cdc-42* in a previous large-scale study [18]. Indeed, when we imaged GFP::WSP-1(GBD) in *vab-1(dx31)* null embryos during intercalation, we observed a significant decrease in the medial enrichment of active CDC-42 (Student’s T-test, p = 0.03; Fig 4B and 4C). These data suggest that VAB-1*/*EphR leads to activation of CDC-42 at the medial edge during dorsal intercalation. We next examined spatial localization of VAB-1 in dorsal epidermal cells using a rescuing, GFP-tagged *vab-1* fosmid. VAB-1::GFP was enriched at medial edges in dorsal cells (S2 Fig) in a manner similar to GFP::WSP-1(GBD).

### Loss of *vab-1/EphR* function phenocopies *cdc-42* loss of function

**Fig 5:**
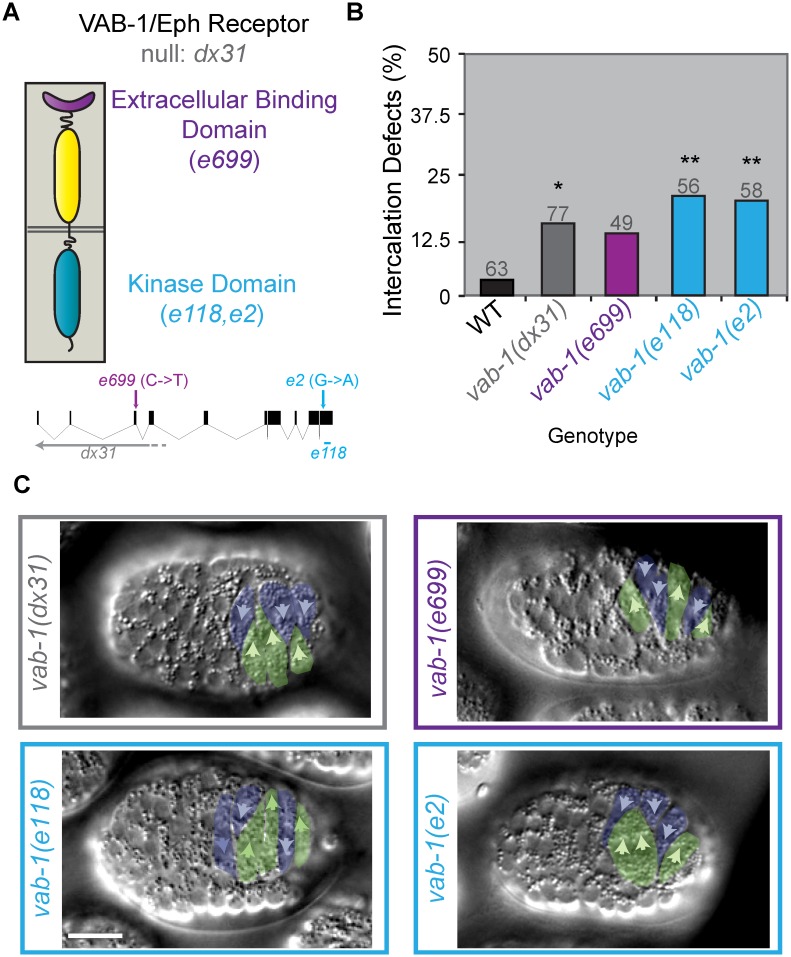
Dorsal intercalation phenotypes in *vab-1/EphR* loss of function embryos. A) Schematic representation of VAB-1/EphR protein structure (top) and *vab-1* alleles (bottom). B) Penetrance of intercalation defects for various *vab-1* alleles. Penetrance was significantly different than wild-type (WT) (* denotes p<0.05, **denotes p<0.007, Fisher’s Exact Test). C) DIC images of representative phenotypes for each *vab-1* allele 90 minutes after terminal division. Scale bar = 10 μm. Gray numbers indicate number of embryos analyzed per genotype.

If VAB-1/EphR is required for medial CDC-42 activity, loss of *vab-1* function would be expected to lead to defects similar to those following loss of *cdc-42* function. When we imaged *vab-1(dx31)* null mutants, we also observed the comigration and medial delay phenotypes (Fig 5; Fisher’s Exact Test to wild-type, p = 0.038) seen in *ZF1*::*cdc-42* and *cdc-42(DN)* mutants. Given the wide array of mapped genetic lesions available within the *vab-1* locus [34], we were additionally able to ask whether certain domains were required for VAB-1 function. We found that animals homozygous for mutations in the kinase domain (*e118* and *e2*), which prevent phosphorylation and activation of VAB-1 [35], also displayed dorsal intercalation defects (Fisher’s Exact Test, p≤0.007). This suggests that, in contrast to some other VAB-1-dependent processes, such as hyp6 cell fusion [35] and amphid axon guidance [36], dorsal intercalation requires VAB-1/EphR kinase activity.

We next examined the potential roles of Eph ligands in dorsal intercalation. First, we examined embryos homozygous for a *vab-1* mutation (*e699*), which causes an amino acid substitution (T63I) within the extracellular domain that binds to canonical ephrin ligands [34, 35, 37, 38]. In contrast to putative kinase-dead mutants, *vab-1(e699)* homozygotes displayed intercalation defects at intermediate levels that were not significantly different from either wild-type or *vab-1(dx31)* null mutants (Fisher’s Exact Test, p = 0.135 and p = 0.796). Given the surprisingly mild defects in *vab-1(e699)* mutants, we next analyzed ephrin mutants directly. Surprisingly, neither triple ephrin mutants (*vab-2(ju1)*, *efn-2(ev658); efn-3(ev696)*) [39] nor putative null mutants of the more divergent ephrin, *efn-4(bx80)* [40], displayed intercalation defects (S3 Fig). Taken together, these data suggest that while kinase activity is important for VAB-1 function during intercalation, interaction with traditional ephrin ligands is dispensable.

### *vab-1/EphR* genetically interacts with *cdc-42* during dorsal intercalation

**Fig 6:**
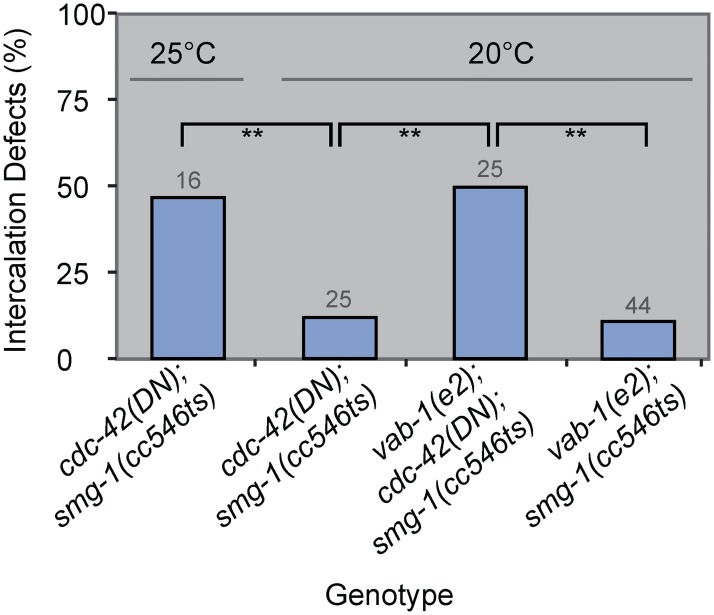
Genetic interaction between *cdc-42* and *vab-1/EphR*. A) Frequency of dorsal intercalation defects in *cdc-42(DN);smg-1(cc546*ts), *cdc-42(DN); vab-1(e2); smg-1(cc546*ts), and *vab-1(e2); smg-1(cc546*ts*)* at indicated temperatures (** denotes p≤0.006, Fisher’s Exact Test). Intercalation defects include ipsilateral comigration and medial delay. Statistical analysis performed with expected values from the additive frequencies of *cdc-42(DN)* 20°C and *vab-1(e2)* was also significantly different than the double loss of function (p<0.04, Fisher’s Exact Test). Gray numbers indicate number of embryos analyzed per genotype.

The phenocopy of *cdc-42* loss of function in *vab-1* mutants, medial enrichment of VAB-1, and the requirement of VAB-1 function for CDC-42 medial enrichment suggests that these two genes lie in the same pathway to promote oriented cell migration during dorsal intercalation. To examine this possibility, we crossed the weak allele, *vab-1(e2)*, into the *cdc-42(DN)* background. We predicted that at a semi-permissive temperature (20°C)—when induction of transgenes using the NMD-sensitive system is weak (16)—the combination of weak *vab-1* and *cdc-42* loss of function would enhance intercalation defects to levels seen at the restrictive temperature (25°C) for *cdc-42(DN)* alone or in *ZF1*::*cdc-42* embryos. Indeed, at 20°C, intercalation defects in *cdc-42(DN)* were significantly less frequent than at 25°C (Fisher’s Exact Test, p = 0.006, Fig 6). Furthermore, the penetrance of defects was significantly enhanced in the *vab-1(e2); cdc-42(DN)* background relative to either *cdc-42(DN)* or *vab-1(e2); smg-1(cc546*ts) grown at 20°C (Fisher’s Exact Test, p≤0.004, Fig 6 and S4 Fig). Moreover, the frequency of enhanced intercalation defects was not significantly different from either *cdc-42(DN)* at 25°C or *ZF1*::*cdc-42*, suggesting that CDC-42 and VAB-1 function together during intercalation. Combined with the loss of medial enrichment of the CDC-42 biosensor in *vab-1(dx31)*, these results suggest that *vab-1* and *cdc-42* function co-linearly in the same genetic pathway during intercalation to promote proper cell guidance and cell migration.

## Discussion

Cell intercalation comprises a series of highly directed cell rearrangements. Given that Cdc42 is a highly conserved regulator of cell guidance, it is surprising that relatively little is known about the role of Cdc42 in this process. While Cdc42 has a known role in regulating adhesion of mesenchymal cells during convergent extension in *Xenopus* [10], there is little evidence for a role for Cdc42 during the intercalation of other cell types, particularly epithelia. Here, using conditional loss of function experiments and an *in vivo* biosensor, we describe a role for CDC-42 at the medial tips of intercalating cells in the dorsal epidermis of *C*. *elegans*. Dorsal intercalation is an epithelial intercalation event that occurs via cell migration and protrusive activity rather than predominantly by spatially-restricted apical junctional rearrangement [15, 16]. Our study suggests that CDC-42 functions with PAR-6, PAR-3, and VAB-1 during dorsal intercalation, particularly through formation and orientation of medial tips.

**Fig 7:**
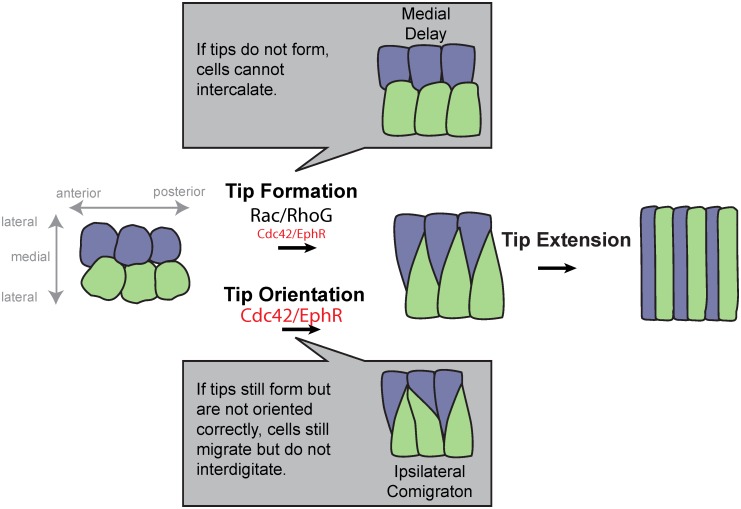
Model of Cdc42’s roles during dorsal intercalation. Posterior intercalating cells in wild-type are shown in the center, with left cells in green and right cells in purple. As the medial tip forms, it must also become oriented. We hypothesize that Cdc42 has roles in both tip formation and orientation (this work, red). Tip formation is highly dependent on Rac/RhoG (large text, see [16]). *cdc-42* and *vab-1* loss of function embryos display blunt medial edges, implicating Cdc42 and EphR in tip formation as well. Ipsilateral comigration in *cdc-42* and *vab-1* loss of function embryos also implicates Cdc42 and EphR in tip orientation. These defects are separable. Even when tip formation is partially defective, cells can still interdigitate; conversely, when tip formation is normal, cells can still comigrate if tips are misoriented.

Loss of *cdc-42* function—either using tissue-specific expression of a dominant-negative (NMD-sensitive system) or degradation of a functional, early maternally-rescuing transgene (ZF1)—can result in intercalating cells that fail to form a medial tip (“medial delay”) and/or that fail to interdigitate properly (“ipsilateral comigration”). Detailed analysis of a cytoplasmic reporter in *ZF1*::*cdc-42* cells uncovered defects in the orientation of medial tips and the extension of dorsal cells. These results suggest that CDC-42 has two roles during dorsal intercalation: 1) to help form and 2) to orient the medial tips of intercalating cells (Fig 7). When cell tips do not form efficiently, the medial edges of cells appear blunt, leading to the medial delay phenotype. These blunt medial edges often resolve eventually, albeit sometimes through comigration of adjacent cells across the dorsal array. When cells do appear to form tips efficiently, they nevertheless often become misoriented, which also leads to comigration of cells that appear otherwise normal.

Since the roles of CDC-42 in tip formation and orientation are separable, we propose two separate roles for CDC-42 during intercalation. Previously, we uncovered a role for *ced-10/Rac* and *mig-2/RhoG* in directing tip formation through actin polymerization mediated by *wve-1/WAVE* and *wsp-1/WASP*, respectively [16]. We hypothesize that CDC-42 plays a minor role in this tip formation process through WASP, which is a well-documented downstream effector of active Cdc42. This hypothesis is consistent with the synergistic effects of weak loss of *ced-10* function combined with perturbation of *cdc-42* function, which specifically affects tip extension.

In addition to its minor role in supporting tip extension in intercalating cells, CDC-42 plays a role in orienting the tips of intercalating cells. We cannot rule out the possibility that subtle orientation defects occur in dorsal epidermal cells prior to the formation of protrusive tips, although as Fig 7 indicates, we favor roles for CDC-42 immediately prior to intercalation. What CDC-42 effector pathways lead to reliable alternation of contralateral cells during dorsal intercalation is an important area of future study. One potential mechanism by which comigration could occur is through abnormal cell adhesion; perhaps adjacent ipsilateral cells adhere too strongly or contralateral cells do not adhere strongly enough in *cdc-42* loss of function embryos. This idea is supported by findings regarding the PAR complex in epithelial cells in *C*. *elegans*: while junctional proteins acquire their normal apicobasal localization in *par-6*::*ZF1* and *par-3*::*ZF1* embryos, they do not form continuous belt-like structures and cells in such embryos display subsequent defects in cell adhesion [8, 9, 24]. While the comigration phenotype has not been reported upon loss of components of the apical junction [41–43], it is possible that inherent local asymmetries of adhesion are required for interdigitation, which would not be revealed by experiments involving simple loss of function. Indeed, the *Drosophila* E-cadherin homologue, Shotgun/DE-cadherin, is asymmetrically localized in the intercalating germband [44], and local junctional disassembly is an important driver of tissue rearrangement [45]. Alternatively, the PAR complex may direct microtubule reorganization (reviewed in [3, 46]) to either form or orient the medial tip. In the single reported drug study, microtubule depolymerization was reported to block intercalation [15] consistent with this possibility. Additional experiments will be required to address these potential mechanisms.

In this work we also describe an upstream role for the sole *C*. *elegans* Eph receptor, VAB-1, in CDC-42 regulation during dorsal intercalation. *vab-1* mutants not only phenocopy *cdc-42* loss of function, but the localization of a biosensor for active CDC-42 to the medial tip is lost in *vab-1* mutants. Nevertheless, the phenotype of *vab-1* null mutants is milder than that resulting from loss of *cdc-42* function. This result suggests that other signaling pathways may impinge on CDC-42 in addition to VAB-1, and warrants further investigation in the future. It is unclear by what mechanism VAB-1 and CDC-42 interact. One straightforward explanation is that VAB-1/EphR activates or localizes a guanine nucleotide exchange factor (GEF) that can activate CDC-42. There are several vertebrate GEFs with such reported specificities: ephexin, intersectin, and Vav2. *ephx-1/*ephexin interacts with EphR to direct Cdc42 activation in neurons in *C*. *elegans* [47, 48]. However, homozygous null *ephx-1* mutants intercalate normally (S5 Fig). Intersectin is a Cdc42 GEF [49, 50] that also binds EphR and WASP [51, 52]. However, the GEF domain is not conserved in worm ITSN-1 [53]. Vav2 is a GEF that mediates endocytosis of Eph-ephrin complexes in neurons [54]. The sole Vav family member in *C*. *elegans*, VAV-1, is required for rhythmic contractions in multiple cell types [55]. Additionally, VAV-1 functions in a pathway with VAB-1 during oocyte maturation [56] without changing VAB-1 localization [39]. A *vav-1* transcriptional reporter [57] is present in the dorsal epidermis [16]. While *vav-1(RNAi)* did not result in intercalation defects [16], it is possible that *vav-1* is functioning redundantly with another GEF during dorsal intercalation.

Another open question regarding VAB-1/EphR is which of its ligands functions during dorsal intercalation. Surprisingly, we found that *vab-1(e699)*, which disrupts the ephrin binding domain, leads to intercalation defects at frequencies between wild-type and *vab-1(dx31)* (Fig 5). However, neither canonical ephrin (*vab-2 efn-2; efn-*3) triple mutants nor a divergent ephrin (*efn-4*) mutant had significant intercalation defects (S3 Fig). There are two possible explanations for these results. First, all four ephrins could function redundantly during dorsal intercalation. Second, dorsal intercalation could be an ephrin-independent process and the *e699* mutation disrupts a portion of the extracellular domain that also binds to other, non-canonical ligands. While the former possibility is difficult to test because EFN-4 has VAB-1 and EFN-1 independent roles that make *vab-1; efn-4* and *efn-1; efn-4* mutants synthetic lethal [40], we sought to investigate the latter possibility. Recently, a noncanonical VAB-1/EphR ligand, VPR-1/VAPB, was identified. VPR-1/VAPB is a secreted protein; mutations in *vpr-1* have pleiotropic effects [58–60]. The progeny of *vpr-1(tm1411)* null homozygotes are maternal effect lethal and display the comigration phenotype (S6 Fig); however, given the widespread disruption of epidermal cell positioning in such embryos we were not able to rule out earlier defects during cell specification and gastrulation as contributing factors. Future studies involving conditional loss of *vpr-1* function will be needed to definitively determine whether *vpr-1* acts in the same pathway as *cdc-42* during dorsal intercalation.

While roles for Cdc42 are well established for migrating cells in culture, roles for Cdc42 during morphogenesis are difficult to establish due to earlier Cdc42-dependent processes, such as gastrulation and polarized cell division. Our results implicate polarized Cdc42 activity in orienting an intercalating epithelium during morphogenesis. Dorsal intercalation is only one of many examples of epithelial intercalation that involve basolateral protrusion [1, 61]. Given its ubiquitous role in establishing cell polarity and in regulating the actin cytoskeleton, it is likely that Cdc42 has widespread functions during these epithelial intercalation events.

## Materials and Methods

### Nematode strains and genetics

Worms were maintained on *Escherichia coli* OP50, as previously described [62]. The wild-type strain used in this study was Bristol N2. Experiments were performed at 20°C unless otherwise specified. The following genetic lesions were utilized in this study. LGI: *par-6(tm1425)*, *smg-1(cc546*ts*)*, *vpr-1(tm1411)*. LGII: *cdc-42(gk388)*, *ephx-1(ok494)*, *vab-1(dx31)*, *vab-1(e699)*, *vab-1(e118)*, *vab-1(e2)*. LGIII: *par-3(it71)*, *unc-119(ed3)*. LGIV: *efn-2(ev658)*, *efn-4(bx80)*, *vab-2(ju1)*. LGX: *efn-3(ev696)*. Additionally, the following transgenic arrays were made for or utilized in this study: *jcEx200[P*_*lin-26*_::*cdc-42(T17N/DN)*::*smg sensitive 3'UTR*, *sur-5*::*mCherry]*, *ojEx99[P*_*cdc-42*_::*gfp*::*wsp-1(G-protein binding domain); unc-119(+)]* based on the *cdc-42* biosensor described in [29], *jcEx273[P*_*cdc-42*_::*gfp*::*wsp-1(G-protein binding domain); P*_*ttx-3*_::*dsRed]*, *jcEx215[P*_*lbp-1*_::*gfp; pRF4]*, *quEx531[vab-1*::*2xTY1*::*gfp*::*3xFLAG* fosmid; *odr-1*::*rfp]*, *ltIs44[P*_*pie-1*_::*PH*_*PLC1∂1*_::*mCherry*, *unc-119(+)]* [30], *reIs9[P*_*lin-26*_::*gfp*::*smg sensitive 3'UTR*, *rol-6(su1006)]* [16], *xnIs83[P*_*cdc-42*_::*2xHA*::*ZF1*::*cdc-42; unc-119(+)]* [22], *zuIs20[P*_*par-3*_::*par-3*::*ZF1*::*gfp; unc-119(+)]* [24], *zuIs43[P*_*pie-1*_::*gfp*::*par-6*::*ZF1; unc-119(+)]* [9].

To make *par-6(M/Z)* mutants, *gfp*::*par-6*::*ZF1; par-6(tm1425)* embryos were obtained through crossing, as described previously [9]. *par-6(M/Z)* mutants die late in embryogenesis [9], so only embryos that died at this stage were analyzed for intercalation defects.

### VAB-1 cloning

The *vab-1* tagged fosmid was obtained through the *C*. *elegans* TransgeneOme project [63]. The *vab-1* fosmid was injected into wild-type animals at 30ng/μL along with 50ng/μL *odr-1*:: *rfp* coinjection marker to obtain transgenic strain IC1403 *quEx531[vab-1*::*2xTY1*::*gfp*::*3xFLAG fosmid; odr-1*::*rfp]*. The *quEx531* array was crossed into *vab-1(dx31)* to create strain IC1487 *vab-1(dx31); quEx531* and tested for functionality.

### Microinjection

To incorporate the epidermally-enriched cytoplasmic reporter, *P*_*lbp-1*_::*gfp*, into *ZF1::cdc-42* worms, we microinjected pSL500[*P*_*lbp-1*_::*gfp*::*unc-54* 3’UTR] [64], directly into the gonads of *ZF1::cdc-42* worms, as described previously [65].

### Crosses

Genetic crosses were used to incorporate the CDC-42 biosensor (gfp::wsp-1(GBD)) into the *vab-1(dx31)* background. To analyze *efn-4(bx80)*, an outcross was performed to remove the *him-5(e1490)* marker from the background. Six outcrosses were performed to remove background mutations from *ephx-1(ok494)* (allele generated in [66]). Additionally, crosses were performed to incorporate *vab-1(e2)* into the CDC-42 dominant-negative and constitutively active backgrounds.

### NMD-dependent conditional expression system

The plasmid backbone (pCM1.3) for the epidermal-specific, NMD-sensitive expression system was described previously [16]. The wild-type *cdc-42* cDNA was amplified with primers DJR491, 5’ TTTTTTTTggccggcctggcATGCAGACGATCAAGTGCGTCGTCG 3’, and DJR512, 5’ TTTTTTcccgggTTAGAGAATATTGCACTTCTTCTTC 3’, digested with *Fse*I and *Xma*I, and cloned into pCM1.3 digested with *Fse*I*/Xma*I to make pCM7.4. Restriction sites in the primers are underlined. The dominant-negative *cdc-42*, *cdc-42(T17N/DN)* (pEWS26), was made with pCM7.4 (*cdc-42(+)*) as a template for PCR site-directed mutagenesis, using the following primers: Forward 5’ ATTGTCTCCTGATCAGCTATACC 3’ Reverse 5’ TTTTACCGACAGCTCCATCTC 3’. Underlined bases denote those mutated relative to wild type.

As described previously [16], gonads of wild-type animals were microinjected with these constructs at 40 ng/μL and a coinjection marker [65]. Resulting lines in the wild-type background were screened for defects using *smg-1* feeding RNAi [67]. Representative lines were crossed into the *smg-1(cc546*ts*)* background, which can be detected by PCR using Forward: 5’ CAGTCGTGAGCTTTGGATGCGTGC 3’ and Reverse: 5’ TCGGGGATACGCAGATTCTTTCCC 3’ followed by digestion specifically of wild-type product using *Msl*I. At least three lines were analyzed per construct. The resulting lines were maintained at 15°C and heat shocked at 25°C for 24 hours to induce transgene expression prior to filming. Crosses were performed at 15°C. Filming was performed at 25°C. For *vab-1(e2)* enhancement and suppression experiments, a semi-permissive temperature of 20°C was used for heat shock and filming.

### DIC imaging and phenotypic scoring

Four dimensional DIC movies were gathered on either a Nikon Optiphot-2 connected to a QImaging camera or Olympus BX50 connected to a Scion camera. ImageJ plugins (available at http://worms.zoology.wisc.edu/research/4d/4d.html) were used to compress and view movies.

Embryos scored “medial delay” appeared to have blunt medial edges for greater than five time points (corresponding to 15 minutes). Embryos were scored “ipsilateral comigration” if at least one pair of two adjacent cells intercalated together across the dorsal array. Some embryos had both defects. For this reason, comigration and medial delay phenotypes are pooled into “intercalation defects”. Though nuclear migration defects were observed in some embryos, they were not considered “intercalation defects” as nuclear migration failure does not prevent successful intercalation [68]. For strains with extrachromosomal arrays—*cdc-42(DN)* and *cdc-42(CA)*—only embryos that inherited the arrays were analyzed, as determined by inheritance of a co-injected nuclear fluorescent protein (SUR-5::mCherry), assayed by epifluorescence. For statistical analysis of intercalation defects among genotypes, Fisher’s Exact Test—a form of chi-squared specialized for low n values that has been used previously to compare *vab-1* alleles [36]—was used.

### Immunostaining

Freeze-cracking was used to permeabilize embryos [69] for antibody staining. Staining was performed as described previously [70]. Embryos were incubated with primary antibodies in PBST+dry milk overnight at 4°C. Embryos were incubated with secondary antibodies in PBST+dry milk for two hours at room temperature. The following primary antibodies were used: 1:1000 rabbit-anti-GFP (Invitrogen), 1:200 mouse-anti-AJM-1 (MH27). The following secondary antibodies were used: 1:50 anti-mouse IgG Texas Red (Jackson ImmunoResearch) and 1:50 anti-rabbit FITC (Jackson ImmunoResearch). Images of stained embryos were acquired as described below.

### Confocal microscopy

Spinning-disk, confocal images were acquired with a Z-slice spacing of 0.4 μm using Micromanager software [71, 72] and a Nikon Eclipse E600 microscope connected to a Yokogawa CSU10 spinning disk scanhead and a Hamamatsu ORCA-ER charge-coupled device (CCD) camera.

#### Medial enrichment

Medial enrichment measures were obtained by calculating the ratio of average intensity measurements at the medial and lateral edges of each cell in ImageJ. Specifically, for each cell, the lasso tool was used to manually select the medial cell edge, lateral cell edge, and a random area in the cytoplasm (non-nuclear) as background. The average intensity of the background was subtracted from the medial and lateral edge measurements prior to calculating the fold enrichment. Projections of 5 focal planes, spaced 0.4 μm apart, were used for this analysis. Only cells that had both a visible medial and lateral edge were analyzed. JMP was used for statistical analysis. For CDC-42 biosensor images, background subtraction and pseudocoloring were performed in ImageJ.

#### Cell morphology

Tip angles were calculated using trigonometric functions as the angle of a line connecting the tip to the cell centroid. The cell centroid, nuclear center, and aspect ratios were measured in ImageJ from the manual outline of each cell/nucleus. PAST was used for angular statistics and graphs [73]. JMP software (SAS; Cary, North Carolina) was used for the remaining statistical analysis.

## Supporting Information

S1 Fig CDC-42 and CED-10/Rac function together during intercalation. A) Dorsal images of *smg-1(cc546)*, *ced-10(n3246)*; *smg-1(cc546)*, dominant-negative *cdc-42(T17N)*, and triple *cdc-42(T17N/DN); ced-10(n3246); smg-1(cc546)* mutants at 25°C. Arrows within cell nuclei point in the direction of migration (left arrows green, right arrows blue). Left-hand cells pseudocolored green, right-hand cells pseudocolored blue. Cells that do not migrate are marked with a white “X”. Scale bar is 10 μm. B) Penetrance of dorsal intercalation defects in *ced-10, cdc-42, smg-1,* double and triple loss-of-function. Perturbation of *ced-10* function significantly increased cdc-42(DN) defects (based on total mount defect frequency, ANOVA, significantly different from all other groups p<0.005).
(TIFF)

S2 Fig *vab-1/*Eph is expressed in the dorsal epidermis. A) Left: α-GFP immunostaining to detect VAB-1::GFP expressed from a rescuing GFP and FLAG-tagged *vab-1* fosmid in posterior intercalating cells. Right: MH27/α-AJM-1 immunostaining in the same embryo. Green arrows denote medial edges. Scale bar = 2.5 μm. B) Quantification of medial/lateral fold enrichment of α-GFP staining relative to MH27/α-AJM-1 staining. Numbers denote number of cells analyzed. * denotes significant difference using Student’s T-test (p = 0.045).
(TIFF)

S3 Fig Dorsal intercalation in ephrin mutants. A) The penetrance of total intercalation defects in neither *vab-2(ju1) efn-2(ev658); efn-3(ev696)* nor *efn-4(bx80)* is significantly different from wild-type (p≥0.50, Fisher’s Exact Test). Gray numbers indicate number of embryos analyzed per genotype. B) Intercalation time in neither *vab-2(ju1) efn-2(ev658); efn-3(ev696)* nor *efn-4(bx80)* is significantly different than wild-type (p≥0.232, Student’s T-test).
(TIFF)

S4 Fig DIC images of dorsal intercalation in *cdc-42(DN); smg-1(cc546*ts*)*, *cdc-42(DN); vab-1(e2); smg-1(cc546*ts), and *vab-1(e2); smg-1(cc546*ts*)* at 20°C. Left dorsal cells pseudocolored green, right cells pseudocolored blue. Yellow dotted lines outline the blunt medial edge. Scale bar = 10 μm.
(TIFF)

S5 Fig Dorsal intercalation phenotypes in *ephx-1/ephexin* embryos. A) The penetrance of intercalation defects in *ephx-1(ok494)* is not significantly different than wild-type (p = 1, Fisher’s Exact Test). Small gray numbers indicate number of embryos analyzed per genotype. B) Intercalation time in *ephx-1(ok494)* is not significantly different than wild-type (p = 0.133, Student’s T-test).
(TIFF)

S6 Fig Maternal effect phenotypes for *vpr-1(tm1411)*. A) DIC images of a *vpr-1(tm1411M/Z)* embryo with intercalation defects. Left dorsal cells pseudocolored green, right cells pseudocolored blue. Light arrows indicate direction of migration. The “0 min.” time point is one hour after the terminal epidermal divisions. Scale bar = 10 μm. B) Many *vpr-1(tm1411M/Z)* die before intercalation. The portion of those remaining that display intercalation defects (~12%) is significantly different than wild-type (WT) (p = 0.01, Fisher’s Exact Test).
(TIFF)

## References

[1] Walck-Shannon E, Hardin J. Cell intercalation from top to bottom. Nature reviews Molecular cell biology. 2014;15(1):34–48. Epub 2013/12/21. 10.1038/nrm3723 . 24355988 PMC4550482

[2] Etienne-Manneville S. Cdc42—the centre of polarity. J Cell Sci. 2004;117(Pt 8):1291–300. 10.1242/jcs.01115 . 15020669

[3] Nobes CD, Hall A. Rho, rac and cdc42 GTPases: regulators of actin structures, cell adhesion and motility. Biochemical Society transactions. 1995;23(3):456–9. . 8566347 10.1042/bst0230456

[4] Rohatgi R, Ma L, Miki H, Lopez M, Kirchhausen T, Takenawa T, et al The interaction between N-WASP and the Arp2/3 complex links Cdc42-dependent signals to actin assembly. Cell. 1999;97(2):221–31. . 10219243 10.1016/s0092-8674(00)80732-1

[5] Guo S, Kemphues KJ. par-1, a gene required for establishing polarity in C. elegans embryos, encodes a putative Ser/Thr kinase that is asymmetrically distributed. Cell. 1995;81(4):611–20. . 7758115 10.1016/0092-8674(95)90082-9

[6] Kay AJ, Hunter CP. CDC-42 regulates PAR protein localization and function to control cellular and embryonic polarity in C. elegans. Curr Biol. 2001;11(7):474–81. . 11412996 10.1016/s0960-9822(01)00141-5

[7] Lin D, Edwards AS, Fawcett JP, Mbamalu G, Scott JD, Pawson T. A mammalian PAR-3-PAR-6 complex implicated in Cdc42/Rac1 and aPKC signalling and cell polarity. Nat Cell Biol. 2000;2(8):540–7. 10.1038/35019582 . 10934475

[8] Achilleos A, Wehman AM, Nance J. PAR-3 mediates the initial clustering and apical localization of junction and polarity proteins during C. elegans intestinal epithelial cell polarization. Development. 2010;137(11):1833–42. Epub 2010/05/01. 10.1242/dev.047647 20431121 PMC2867319

[9] Totong R, Achilleos A, Nance J. PAR-6 is required for junction formation but not apicobasal polarization in C. elegans embryonic epithelial cells. Development. 2007;134(7):1259–68. Epub 2007/02/23. 10.1242/dev.02833 . 17314130

[10] Choi SC, Han JK. Xenopus Cdc42 regulates convergent extension movements during gastrulation through Wnt/Ca2+ signaling pathway. Dev Biol. 2002;244(2):342–57. 10.1006/dbio.2002.0602 . 11944942

[11] Oda-Ishii I, Ishii Y, Mikawa T. Eph regulates dorsoventral asymmetry of the notochord plate and convergent extension-mediated notochord formation. PLoS One. 2010;5(10):e13689 10.1371/journal.pone.0013689 21060822 PMC2966392

[12] Clark IB, Muha V, Klingseisen A, Leptin M, Muller HA. Fibroblast growth factor signalling controls successive cell behaviours during mesoderm layer formation in Drosophila. Development. 2011;138(13):2705–15. 10.1242/dev.060277 21613323 PMC3109598

[13] Reymond N, Im JH, Garg R, Vega FM, Borda d'Agua B, Riou P, et al Cdc42 promotes transendothelial migration of cancer cells through beta1 integrin. J Cell Biol. 2012;199(4):653–68. 10.1083/jcb.201205169 23148235 PMC3494849

[14] Kieserman EK, Wallingford JB. In vivo imaging reveals a role for Cdc42 in spindle positioning and planar orientation of cell divisions during vertebrate neural tube closure. J Cell Sci. 2009;122(Pt 14):2481–90. 10.1242/jcs.042135 19549689 PMC2704883

[15] Williams-Masson EM, Heid PJ, Lavin CA, Hardin J. The cellular mechanism of epithelial rearrangement during morphogenesis of the Caenorhabditis elegans dorsal hypodermis. Dev Biol. 1998;204(1):263–76. 10.1006/dbio.1998.9048 9851858

[16] Walck-Shannon E, Reiner D, Hardin J. Polarized Rac-dependent protrusions drive epithelial intercalation in the embryonic epidermis of C. elegans. Development. 2015;142(20):3549–60. 10.1242/dev.127597 26395474 PMC4631769

[17] Ouellette MH, Martin E, Lacoste-Caron G, Hamiche K, Jenna S. Spatial control of active CDC-42 during collective migration of hypodermal cells in Caenorhabditis elegans. J Mol Cell Biol. 2016;8(4):313–27. 10.1093/jmcb/mjv062 . 26578656 PMC4991665

[18] Lehner B, Crombie C, Tischler J, Fortunato A, Fraser AG. Systematic mapping of genetic interactions in Caenorhabditis elegans identifies common modifiers of diverse signaling pathways. Nat Genet. 2006;38(8):896–903. 10.1038/ng1844 . 16845399

[19] Raftopoulou M, Hall A. Cell migration: Rho GTPases lead the way. Dev Biol. 2004;265(1):23–32. . 14697350 10.1016/j.ydbio.2003.06.003

[20] Gotta M, Abraham MC, Ahringer J. CDC-42 controls early cell polarity and spindle orientation in C. elegans. Curr Biol. 2001;11(7):482–8. . 11412997 10.1016/s0960-9822(01)00142-7

[21] Bourne HR, Sanders DA, McCormick F. The GTPase superfamily: conserved structure and molecular mechanism. Nature. 1991;349(6305):117–27. 10.1038/349117a0 . 1898771

[22] Armenti ST, Lohmer LL, Sherwood DR, Nance J. Repurposing an endogenous degradation system for rapid and targeted depletion of C. elegans proteins. Development. 2014;141(23):4640–7. 10.1242/dev.115048 25377555 PMC4302935

[23] Chan E, Nance J. Mechanisms of CDC-42 activation during contact-induced cell polarization. J Cell Sci. 2013;126(Pt 7):1692–702. 10.1242/jcs.124594 23424200 PMC3647442

[24] Nance J, Munro EM, Priess JR. C. elegans PAR-3 and PAR-6 are required for apicobasal asymmetries associated with cell adhesion and gastrulation. Development. 2003;130(22):5339–50. 10.1242/dev.00735 . 13129846

[25] Neukomm LJ, Zeng S, Frei AP, Huegli PA, Hengartner MO. Small GTPase CDC-42 promotes apoptotic cell corpse clearance in response to PAT-2 and CED-1 in C. elegans. Cell Death Differ. 2014;21(6):845–53. 10.1038/cdd.2014.23 24632947 PMC4013519

[26] Welchman DP, Mathies LD, Ahringer J. Similar requirements for CDC-42 and the PAR-3/PAR-6/PKC-3 complex in diverse cell types. Dev Biol. 2007;305(1):347–57. 10.1016/j.ydbio.2007.02.022 17383625 PMC3330270

[27] Anderson DC, Gill JS, Cinalli RM, Nance J. Polarization of the C. elegans embryo by RhoGAP-mediated exclusion of PAR-6 from cell contacts. Science. 2008;320(5884):1771–4. 10.1126/science.1156063 18583611 PMC2670547

[28] Plenefisch J, Xiao H, Mei B, Geng J, Komuniecki PR, Komuniecki R. Secretion of a novel class of iFABPs in nematodes: coordinate use of the Ascaris/Caenorhabditis model systems. Mol Biochem Parasitol. 2000;105(2):223–36. . 10693745 10.1016/s0166-6851(99)00179-6

[29] Kumfer KT, Cook SJ, Squirrell JM, Eliceiri KW, Peel N, O'Connell KF, et al CGEF-1 and CHIN-1 regulate CDC-42 activity during asymmetric division in the Caenorhabditis elegans embryo. Mol Biol Cell. 2010;21(2):266–77. 10.1091/mbc.E09-01-0060 19923324 PMC2808230

[30] Kachur TM, Audhya A, Pilgrim DB. UNC-45 is required for NMY-2 contractile function in early embryonic polarity establishment and germline cellularization in C. elegans. Dev Biol. 2008;314(2):287–99. 10.1016/j.ydbio.2007.11.028 . 18190904

[31] Atwood SX, Chabu C, Penkert RR, Doe CQ, Prehoda KE. Cdc42 acts downstream of Bazooka to regulate neuroblast polarity through Par-6 aPKC. J Cell Sci. 2007;120(Pt 18):3200–6. 10.1242/jcs.014902 17726059 PMC1988841

[32] Motegi F, Seydoux G. The PAR network: redundancy and robustness in a symmetry-breaking system. Philos Trans R Soc Lond B Biol Sci. 2013;368(1629):20130010 10.1098/rstb.2013.0010 24062581 PMC3785961

[33] Ikegami R, Simokat K, Zheng H, Brown L, Garriga G, Hardin J, et al Semaphorin and Eph receptor signaling guide a series of cell movements for ventral enclosure in C. elegans. Curr Biol. 2012;22(1):1–11. 10.1016/j.cub.2011.12.009 22197242 PMC4306670

[34] George SE, Simokat K, Hardin J, Chisholm AD. The VAB-1 Eph receptor tyrosine kinase functions in neural and epithelial morphogenesis in C. elegans. Cell. 1998;92(5):633–43. . 9506518 10.1016/s0092-8674(00)81131-9

[35] Wang X, Roy PJ, Holland SJ, Zhang LW, Culotti JG, Pawson T. Multiple ephrins control cell organization in C. elegans using kinase-dependent and -independent functions of the VAB-1 Eph receptor. Mol Cell. 1999;4(6):903–13. . 10635316 10.1016/s1097-2765(00)80220-8

[36] Grossman EN, Giurumescu CA, Chisholm AD. Mechanisms of ephrin receptor protein kinase-independent signaling in amphid axon guidance in Caenorhabditis elegans. Genetics. 2013;195(3):899–913. 10.1534/genetics.113.154393 23979582 PMC3813872

[37] Chin-Sang ID, George SE, Ding M, Moseley SL, Lynch AS, Chisholm AD. The ephrin VAB-2/EFN-1 functions in neuronal signaling to regulate epidermal morphogenesis in C. elegans. Cell. 1999;99(7):781–90. . 10619431 10.1016/s0092-8674(00)81675-x

[38] Himanen JP, Henkemeyer M, Nikolov DB. Crystal structure of the ligand-binding domain of the receptor tyrosine kinase EphB2. Nature. 1998;396(6710):486–91. 10.1038/24904 . 9853759

[39] Cheng H, Govindan JA, Greenstein D. Regulated trafficking of the MSP/Eph receptor during oocyte meiotic maturation in C. elegans. Curr Biol. 2008;18(10):705–14. 10.1016/j.cub.2008.04.043 18472420 PMC2613949

[40] Chin-Sang ID, Moseley SL, Ding M, Harrington RJ, George SE, Chisholm AD. The divergent C. elegans ephrin EFN-4 functions inembryonic morphogenesis in a pathway independent of the VAB-1 Eph receptor. Development. 2002;129(23):5499–510. . 12403719 10.1242/dev.00122

[41] Costa M, Raich W, Agbunag C, Leung B, Hardin J, Priess JR. A putative catenin-cadherin system mediates morphogenesis of the Caenorhabditis elegans embryo. J Cell Biol. 1998;141(1):297–308. 9531567 10.1083/jcb.141.1.297 PMC2132712

[42] Koppen M, Simske JS, Sims PA, Firestein BL, Hall DH, Radice AD, et al Cooperative regulation of AJM-1 controls junctional integrity in Caenorhabditis elegans epithelia. Nat Cell Biol. 2001;3(11):983–91. 10.1038/ncb1101-983 . 11715019

[43] Simske JS, Koppen M, Sims P, Hodgkin J, Yonkof A, Hardin J. The cell junction protein VAB-9 regulates adhesion and epidermal morphology in C. elegans. Nat Cell Biol. 2003;5(7):619–25. 10.1038/ncb1002 . 12819787

[44] Blankenship JT, Backovic ST, Sanny JS, Weitz O, Zallen JA. Multicellular rosette formation links planar cell polarity to tissue morphogenesis. Dev Cell. 2006;11(4):459–70. 10.1016/j.devcel.2006.09.007 . 17011486

[45] Tamada M, Farrell DL, Zallen JA. Abl regulates planar polarized junctional dynamics through beta-catenin tyrosine phosphorylation. Dev Cell. 2012;22(2):309–19. 10.1016/j.devcel.2011.12.025 22340496 PMC3327890

[46] Etienne-Manneville S. Microtubules in cell migration. Annu Rev Cell Dev Biol. 2013;29:471–99. 10.1146/annurev-cellbio-101011-155711 . 23875648

[47] Frank CA, Pielage J, Davis GW. A presynaptic homeostatic signaling system composed of the Eph receptor, ephexin, Cdc42, and CaV2.1 calcium channels. Neuron. 2009;61(4):556–69. 10.1016/j.neuron.2008.12.028 19249276 PMC2699049

[48] Shamah SM, Lin MZ, Goldberg JL, Estrach S, Sahin M, Hu L, et al EphA receptors regulate growth cone dynamics through the novel guanine nucleotide exchange factor ephexin. Cell. 2001;105(2):233–44. . 11336673 10.1016/s0092-8674(01)00314-2

[49] Pruitt WM, Karnoub AE, Rakauskas AC, Guipponi M, Antonarakis SE, Kurakin A, et al Role of the pleckstrin homology domain in intersectin-L Dbl homology domain activation of Cdc42 and signaling. Biochim Biophys Acta. 2003;1640(1):61–8. . 12676355 10.1016/s0167-4889(03)00002-8

[50] Zamanian JL, Kelly RB. Intersectin 1L guanine nucleotide exchange activity is regulated by adjacent src homology 3 domains that are also involved in endocytosis. Mol Biol Cell. 2003;14(4):1624–37. 10.1091/mbc.E02-08-0494 12686614 PMC153127

[51] Irie F, Yamaguchi Y. EphB receptors regulate dendritic spine development via intersectin, Cdc42 and N-WASP. Nat Neurosci. 2002;5(11):1117–8. 10.1038/nn964 . 12389031

[52] Hussain NK, Jenna S, Glogauer M, Quinn CC, Wasiak S, Guipponi M, et al Endocytic protein intersectin-l regulates actin assembly via Cdc42 and N-WASP. Nat Cell Biol. 2001;3(10):927–32. 10.1038/ncb1001-927 . 11584276

[53] Rose S, Malabarba MG, Krag C, Schultz A, Tsushima H, Di Fiore PP, et al Caenorhabditis elegans intersectin: a synaptic protein regulating neurotransmission. Mol Biol Cell. 2007;18(12):5091–9. 10.1091/mbc.E07-05-0460 17942601 PMC2096573

[54] Cowan CW, Shao YR, Sahin M, Shamah SM, Lin MZ, Greer PL, et al Vav family GEFs link activated Ephs to endocytosis and axon guidance. Neuron. 2005;46(2):205–17. 10.1016/j.neuron.2005.03.019 . 15848800

[55] Norman KR, Fazzio RT, Mellem JE, Espelt MV, Strange K, Beckerle MC, et al The Rho/Rac-family guanine nucleotide exchange factor VAV-1 regulates rhythmic behaviors in C. elegans. Cell. 2005;123(1):119–32. 10.1016/j.cell.2005.08.001 . 16213217

[56] Govindan JA, Cheng H, Harris JE, Greenstein D. Galphao/i and Galphas signaling function in parallel with the MSP/Eph receptor to control meiotic diapause in C. elegans. Curr Biol. 2006;16(13):1257–68. 10.1016/j.cub.2006.05.020 . 16824915

[57] Ziel JW, Matus DQ, Sherwood DR. An expression screen for RhoGEF genes involved in C. elegans gonadogenesis. Gene Expr Patterns. 2009;9(6):397–403. 10.1016/j.gep.2009.06.005 19540360 PMC2726742

[58] Tsuda H, Han SM, Yang Y, Tong C, Lin YQ, Mohan K, et al The amyotrophic lateral sclerosis 8 protein VAPB is cleaved, secreted, and acts as a ligand for Eph receptors. Cell. 2008;133(6):963–77. 10.1016/j.cell.2008.04.039 18555774 PMC2494862

[59] Han SM, Tsuda H, Yang Y, Vibbert J, Cottee P, Lee SJ, et al Secreted VAPB/ALS8 major sperm protein domains modulate mitochondrial localization and morphology via growth cone guidance receptors. Dev Cell. 2012;22(2):348–62. 10.1016/j.devcel.2011.12.009 22264801 PMC3298687

[60] Han SM, El Oussini H, Scekic-Zahirovic J, Vibbert J, Cottee P, Prasain JK, et al VAPB/ALS8 MSP ligands regulate striated muscle energy metabolism critical for adult survival in caenorhabditis elegans. PLoS Genet. 2013;9(9):e1003738 10.1371/journal.pgen.1003738 24039594 PMC3764199

[61] Williams M, Yen W, Lu X, Sutherland A. Distinct apical and basolateral mechanisms drive planar cell polarity-dependent convergent extension of the mouse neural plate. Dev Cell. 2014;29(1):34–46. 10.1016/j.devcel.2014.02.007 24703875 PMC4120093

[62] Brenner S. The genetics of Caenorhabditis elegans. Genetics. 1974;77(1):71–94. 4366476 10.1093/genetics/77.1.71 PMC1213120

[63] Sarov M, Murray JI, Schanze K, Pozniakovski A, Niu W, Angermann K, et al A genome-scale resource for in vivo tag-based protein function exploration in C. elegans. Cell. 2012;150(4):855–66. 10.1016/j.cell.2012.08.001 22901814 PMC3979301

[64] Fridolfsson HN, Starr DA. Kinesin-1 and dynein at the nuclear envelope mediate the bidirectional migrations of nuclei. J Cell Biol. 2010;191(1):115–28. 10.1083/jcb.201004118 20921138 PMC2953438

[65] Mello C, Fire A. DNA transformation. Methods Cell Biol. 1995;48:451–82. . 8531738

[66] Consortium CeDM. Large-scale screening for targeted knockouts in the Caenorhabditis elegans genome. G3 (Bethesda). 2012;2(11):1415–25. 10.1534/g3.112.003830 23173093 PMC3484672

[67] Kamath RS, Fraser AG, Dong Y, Poulin G, Durbin R, Gotta M, et al Systematic functional analysis of the Caenorhabditis elegans genome using RNAi. Nature. 2003;421(6920):231–7. 10.1038/nature01278 . 12529635

[68] Malone CJ, Fixsen WD, Horvitz HR, Han M. UNC-84 localizes to the nuclear envelope and is required for nuclear migration and anchoring during C. elegans development. Development. 1999;126(14):3171–81. . 10375507 10.1242/dev.126.14.3171

[69] Albertson DG. Formation of the first cleavage spindle in nematode embryos. Dev Biol. 1984;101(1):61–72. . 6692980 10.1016/0012-1606(84)90117-9

[70] Leung B, Hermann GJ, Priess JR. Organogenesis of the Caenorhabditis elegans intestine. Dev Biol. 1999;216(1):114–34. 10.1006/dbio.1999.9471 . 10588867

[71] Edelstein AD, Tsuchida MA, Amodaj N, Pinkard H, Vale RD, Stuurman N. Advanced methods of microscope control using μManager software. J Biol Methods. 2014;1(2). 10.14440/jbm.2014.36 25606571 PMC4297649

[72] Edelstein A, Amodaj N, Hoover K, Vale R, Stuurman N. Computer control of microscopes using microManager. Curr Protoc Mol Biol. 2010;Chapter 14:Unit14 20 10.1002/0471142727.mb1420s92 20890901 PMC3065365

[73] Hammer Ø, Harper DAT, Ryan PD. PAST: Paleontological statistics software package for education and data analysis. Palaeontol Electron. 2001;4(1):9pp.

