# Supplementary figures and images for: CDC-42 Orients Cell Migration during Epithelial Intercalation in the Caenorhabditis elegans Epidermis
Source: PLoS Genet. 2016 Nov 18;12(11):e1006415. doi: 10.1371/journal.pgen.1006415 (PMC5127194; doi:10.1371/journal.pgen.1006415)

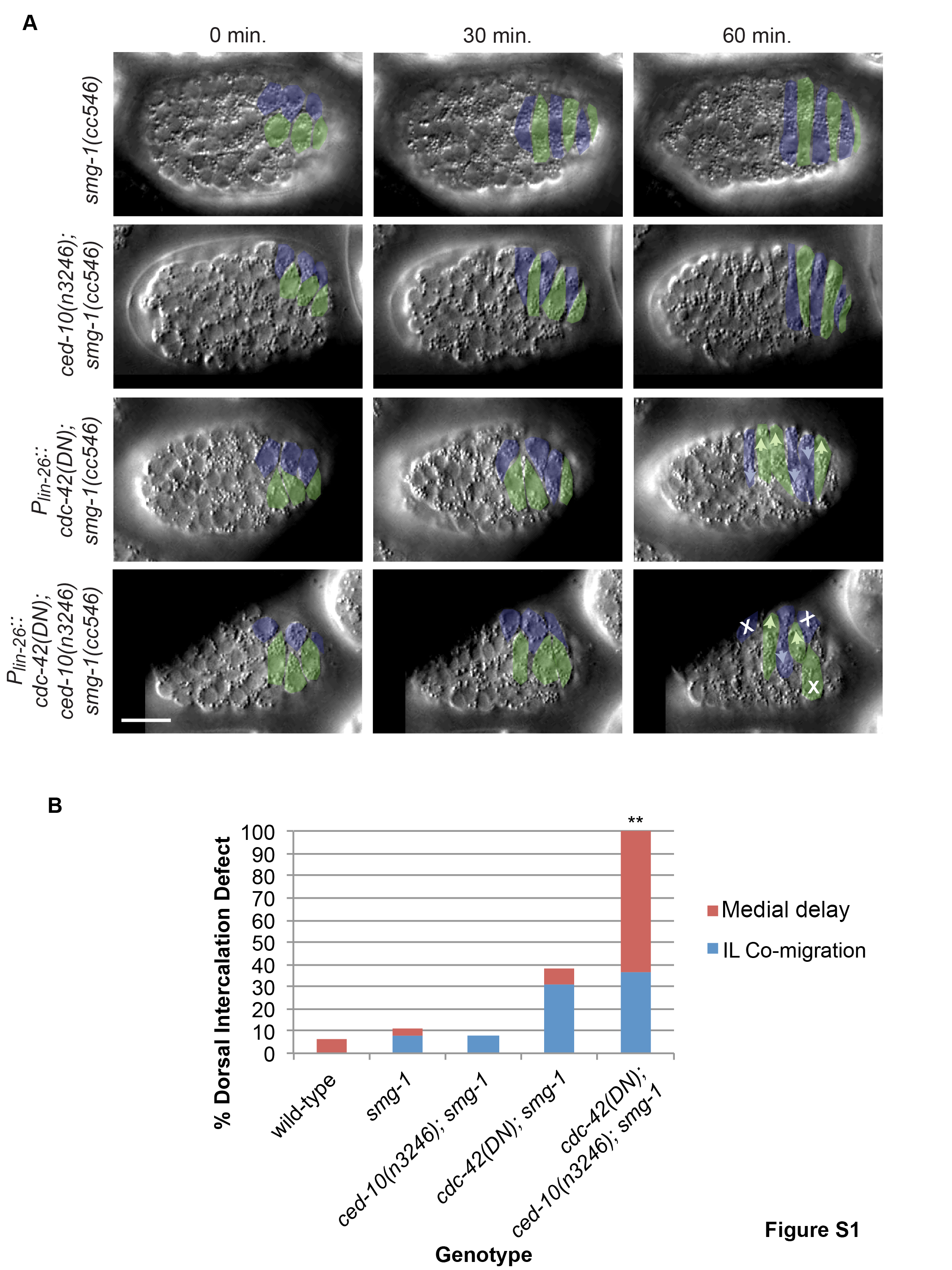

Supplement: S1 Fig — A) Dorsal images of smg-1(cc546), ced-10(n3246); smg-1(cc546), dominant-negative cdc-42(T17N), and triple cdc-42(T17N/DN); ced-10(n3246); smg-1(cc546) mutants at 25°C. Arrows within cell nuclei point in the direction of migration (left arrows green, right arrows blue). Left-hand cells pseudocolored green, right-hand cells pseudocolored blue. Cells that do not migrate are marked with a white “X”. Scale bar is 10 μm. B) Penetrance of dorsal intercalation defects in ced-10, cdc-42, smg-1, double and triple loss-of-function. Perturbation of ced-10 function significantly increased cdc-42(DN) defects (based on total mount defect frequency, ANOVA, significantly different from all other groups p<0.005). (TIFF) [file pgen.1006415.s001.tiff]

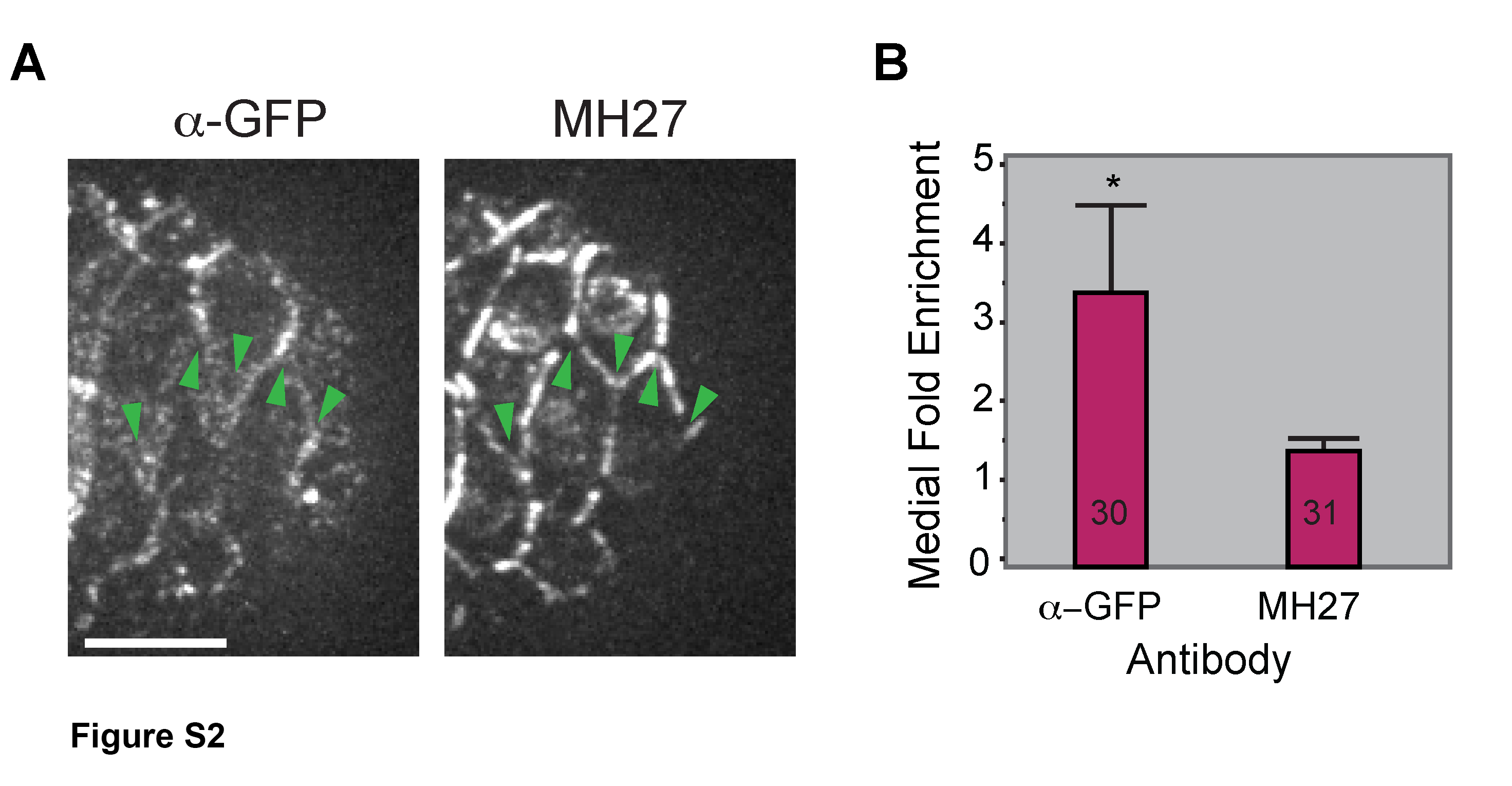

Supplement: S2 Fig — A) Left: α-GFP immunostaining to detect VAB-1::GFP expressed from a rescuing GFP and FLAG-tagged vab-1 fosmid in posterior intercalating cells. Right: MH27/α-AJM-1 immunostaining in the same embryo. Green arrows denote medial edges. Scale bar = 2.5 μm. B) Quantification of medial/lateral fold enrichment of α-GFP staining relative to MH27/α-AJM-1 staining. Numbers denote number of cells analyzed. * denotes significant difference using Student’s T-test (p = 0.045). (TIFF) [file pgen.1006415.s002.tiff]

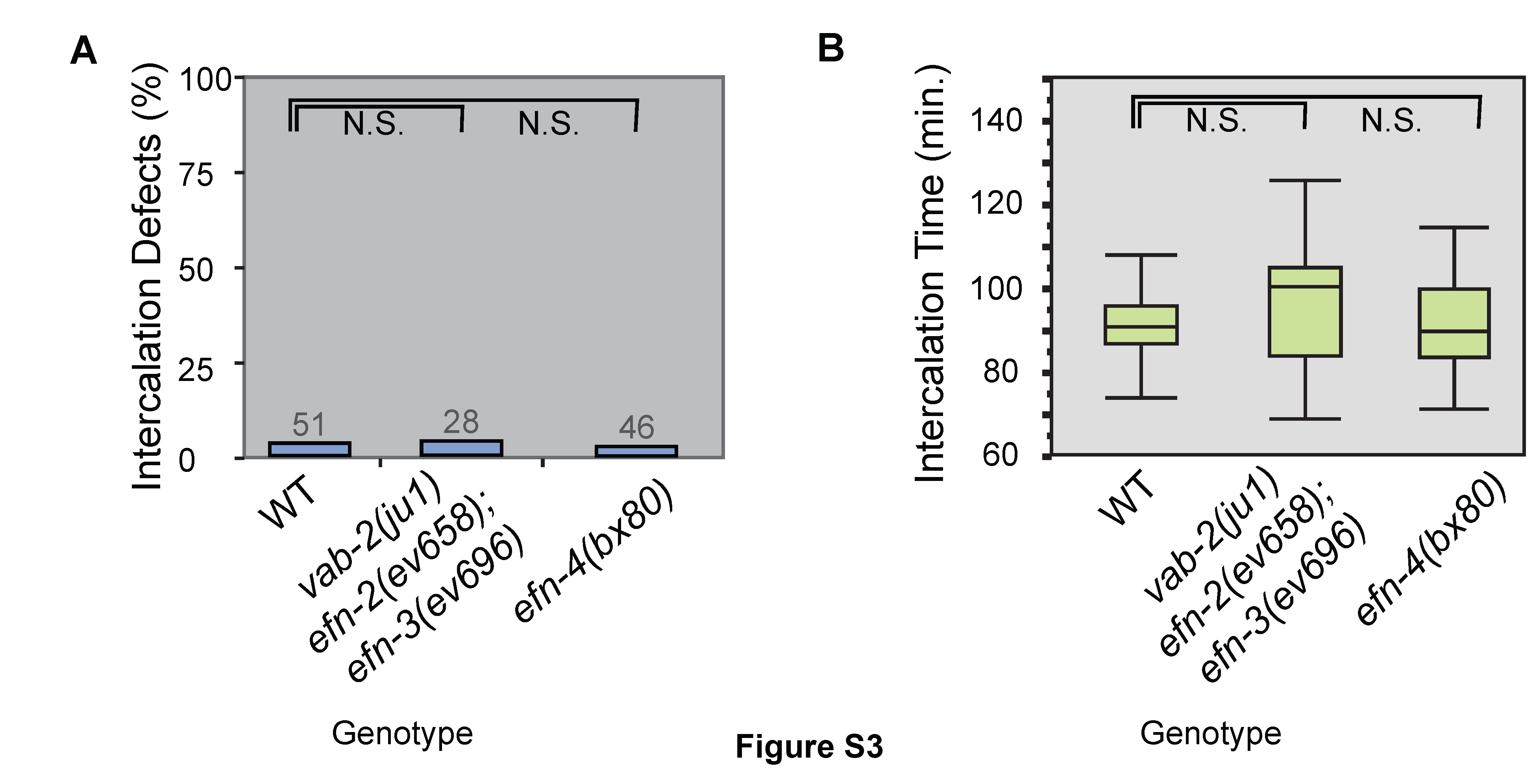

Supplement: S3 Fig — A) The penetrance of total intercalation defects in neither vab-2(ju1) efn-2(ev658); efn-3(ev696) nor efn-4(bx80) is significantly different from wild-type (p≥0.50, Fisher’s Exact Test). Gray numbers indicate number of embryos analyzed per genotype. B) Intercalation time in neither vab-2(ju1) efn-2(ev658); efn-3(ev696) nor efn-4(bx80) is significantly different than wild-type (p≥0.232, Student’s T-test). (TIFF) [file pgen.1006415.s003.tiff]

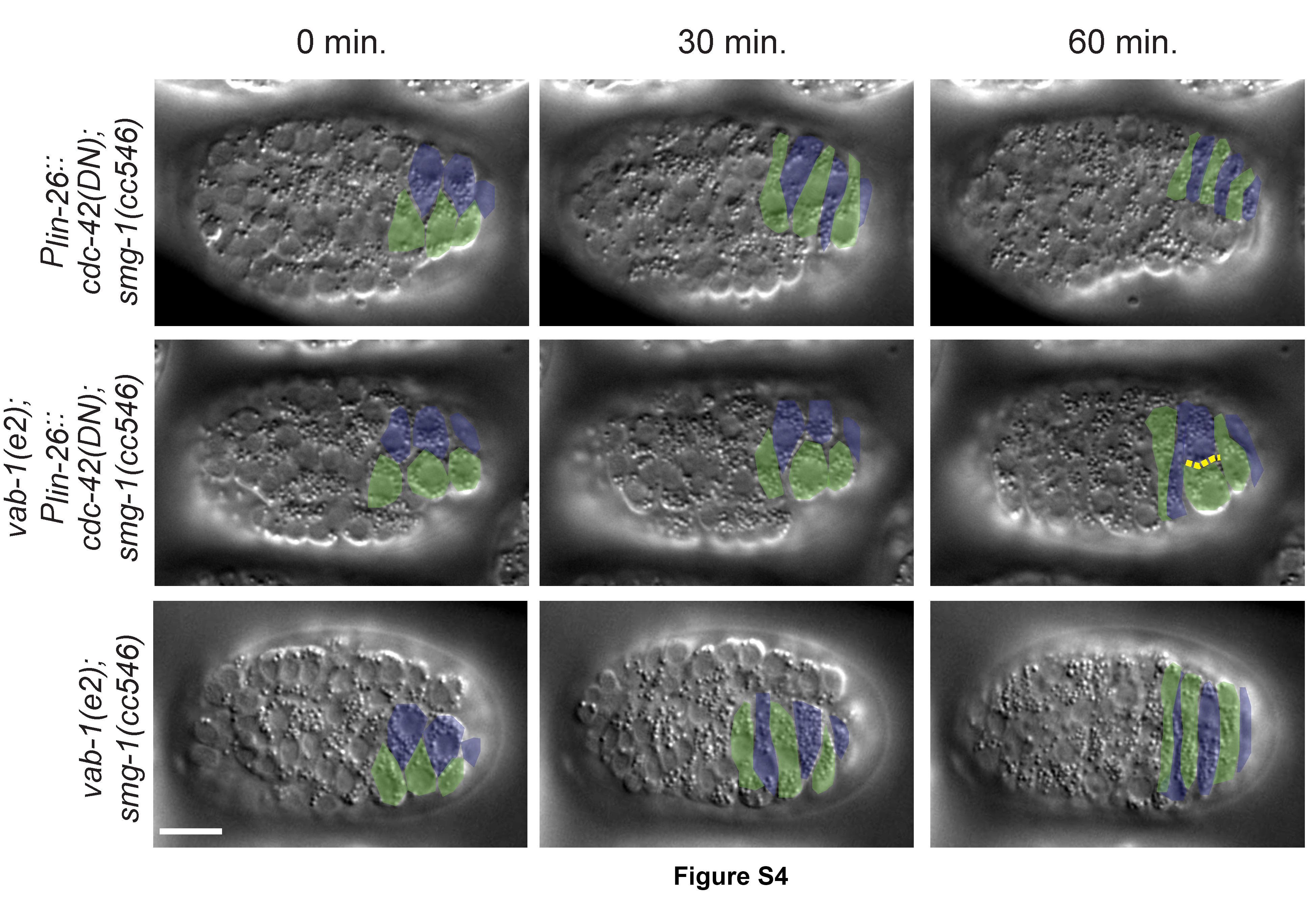

Supplement: S4 Fig — Left dorsal cells pseudocolored green, right cells pseudocolored blue. Yellow dotted lines outline the blunt medial edge. Scale bar = 10 μm. (TIFF) [file pgen.1006415.s004.tiff]

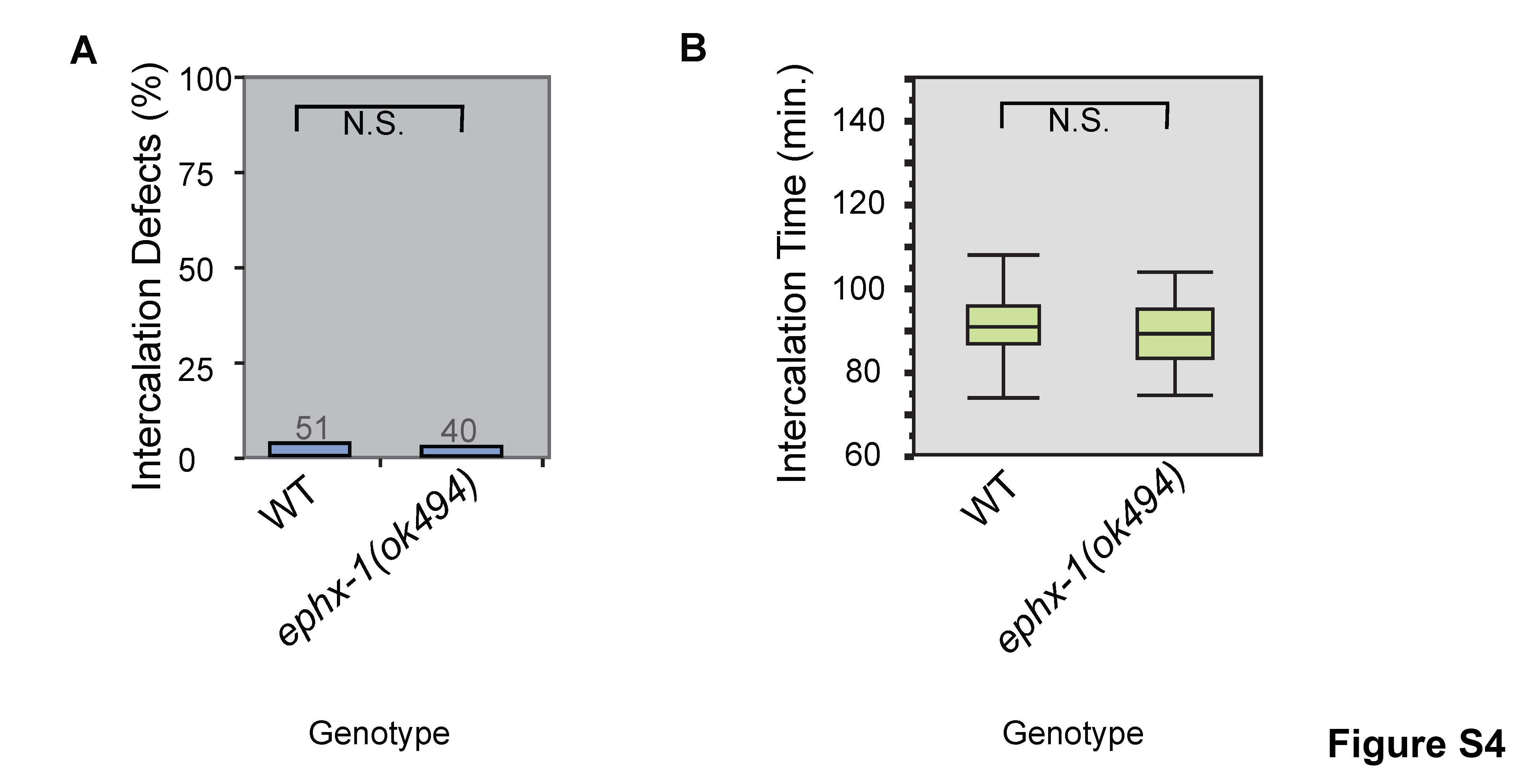

Supplement: S5 Fig — A) The penetrance of intercalation defects in ephx-1(ok494) is not significantly different than wild-type (p = 1, Fisher’s Exact Test). Small gray numbers indicate number of embryos analyzed per genotype. B) Intercalation time in ephx-1(ok494) is not significantly different than wild-type (p = 0.133, Student’s T-test). (TIFF) [file pgen.1006415.s005.tiff]

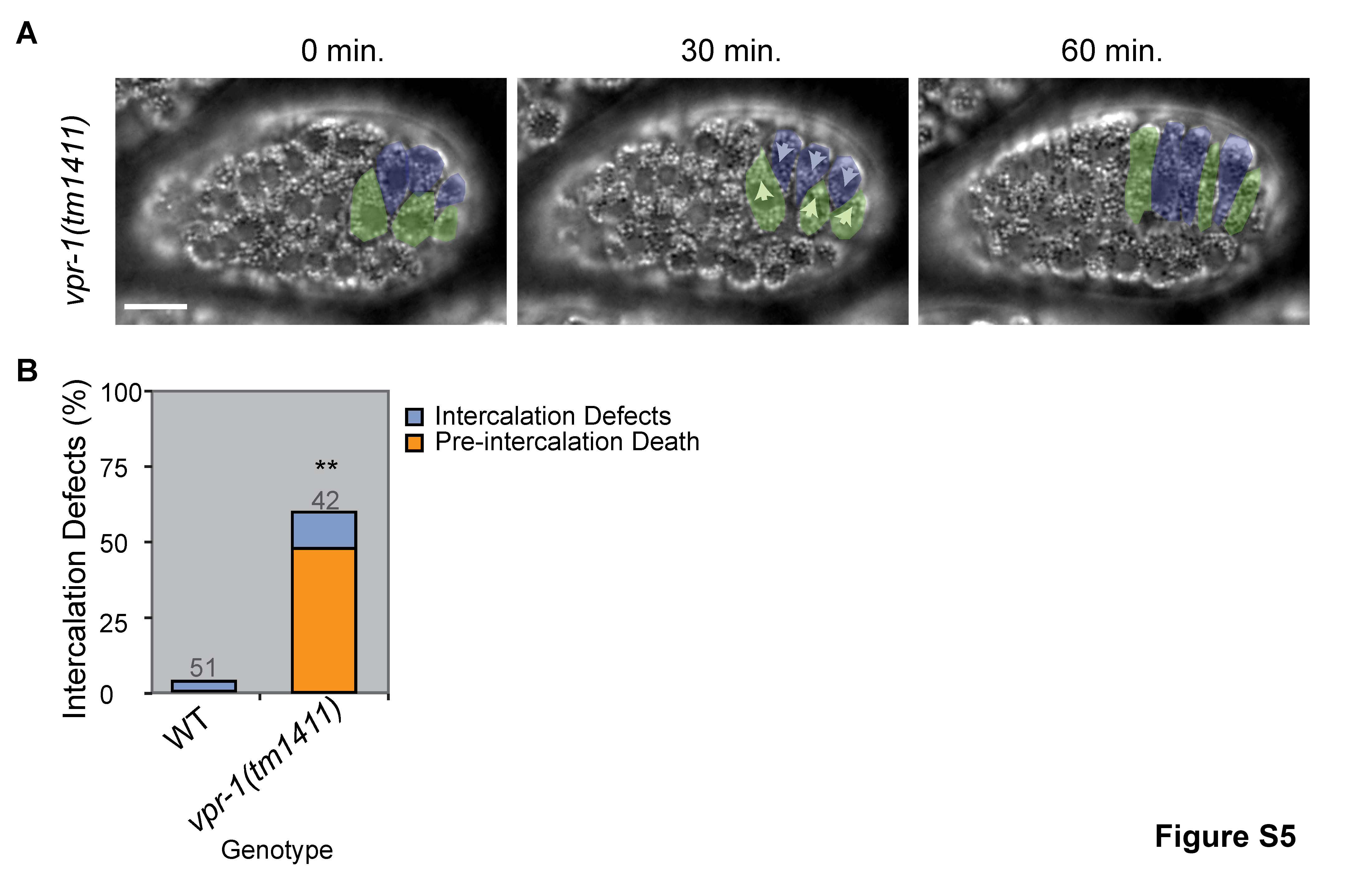

Supplement: S6 Fig — A) DIC images of a vpr-1(tm1411M/Z) embryo with intercalation defects. Left dorsal cells pseudocolored green, right cells pseudocolored blue. Light arrows indicate direction of migration. The “0 min.” time point is one hour after the terminal epidermal divisions. Scale bar = 10 μm. B) Many vpr-1(tm1411M/Z) die before intercalation. The portion of those remaining that display intercalation defects (~12%) is significantly different than wild-type (WT) (p = 0.01, Fisher’s Exact Test). (TIFF) [file pgen.1006415.s006.tiff]
